# The Native Dietary Habits of the Two Sympatric Bee Species and Their Effects on Shaping Midgut Microorganisms

**DOI:** 10.3389/fmicb.2021.738226

**Published:** 2021-10-07

**Authors:** Ying Wang, Zhenfang Li, Lanting Ma, Guilin Li, Kai Han, Zhenguo Liu, Hongfang Wang, Baohua Xu

**Affiliations:** ^1^Department of Science and Technology, Shandong Agricultural University, Taian, China; ^2^College of Plant Protection, China Agricultural University, Beijing, China; ^3^College of Animal Science and Technology, Shandong Agricultural University, Taian, China; ^4^College of Life Sciences, Qufu Normal University, Jining, China

**Keywords:** dietary habits, honeybee, intestinal microorganism, hypopharyngeal gland, digestive enzyme

## Abstract

The intestinal microbial community composition of different bee species typically has host specificity, yet little is known about the underlying formation mechanism. There are signs that dietary habits vary in different bee species, suggesting that there may be close relationships between dietary habits and intestinal microorganisms. We explored this hypothesis by comparing the dietary habits and gut microbiota of two common bee species (*Apis mellifera* L. and *Apis cerana cerana*) in China. Bee bread and midgut samples from wild and laboratory-reared bees were collected, and the differences in intestinal microbial community composition and growth and development before and after the change in dietary habits of different bee species were compared. We found that the two sympatric species had different dietary specializations and similar metagenomic diversities. The microbiota composition differed between the two species. Moreover, we revealed that changes in native dietary habits destroyed the intestinal microbiota community composition, negatively affecting the growth and development of honeybees.

## Introduction

As a large microbiological body, the diet and gut microbiome have fundamental roles in host nutrition, physiology, and health (Clemente et al., 2012; Chatelier et al., 2013; Kartzinel et al., 2019). Studies on the intestinal microbial communities of different hosts have shown that the composition of the microbial community is very different among different hosts (Burke et al., 2011; David et al., 2014; Kartzinel et al., 2019), and the intestinal microbial functional structure of specific species has high stability and spatiotemporal specificity (Antonopoulos et al., 2010; Caporaso et al., 2011; Martiny et al., 2015; Oh et al., 2016; Goldford et al., 2018). Mounting evidence suggests that gut microbiome composition and diversity are mainly affected by environmental factors, and dietary habits (especially dietary structure) may play a critical role in shaping the host gut microbiome (Falony et al., 2016; Zhernakova et al., 2016; Rothschild et al., 2018; Kartzinel et al., 2019). Recently, a study on food and human microbiomes first provided evidence that fermented foods can be a possible source of *lactic acid* bacteria for the human gut microbiome (Pasolli et al., 2020). A previous study also found that insect microbiomes depend on the microbiomes in their living environment (Hannula et al., 2019). However, it is still not fully understood how diet interacts with the host gut microbiome.

Honeybees are a good model for microbiome research (Engel et al., 2016). Honeybees are one of the few animals that can create fermented food, such as honey and bee bread. Bee bread contains approximately 15–28% proteins, 8–10% moisture, 3–9% lipids, 24–35% carbohydrates, 3–5% minerals (Bleha et al., 2019; Khalifa et al., 2020), and a variety of bacteria (Anderson et al., 2013; Disayathanoowat et al., 2020) and fungal communities (Disayathanoowat et al., 2020), and the nutrient composition of bee bread varies with different plant sources (Mayda et al., 2020), bee species (Capcarova et al., 2019), and production areas (Sobral et al., 2017; Urcan et al., 2018; Bleha et al., 2019). Some evidence shows that the nutritional components of bee pollen change significantly during the process of transformation into bee bread (DeGrandi-Hoffman et al., 2013; Mayda et al., 2020). The composition of gut microbiota among the same bee species is highly conserved and specific (Kwong and Moran, 2016; Raymann and Moran, 2018), but considerable variation in gut communities has been found among bee species (Kwong et al., 2017). However, the related formation mechanism is still unclear.

A recent study of two commercial species of honeybees under the same environmental conditions showed similar core microbial community structures in bee pollen and bee breads (Disayathanoowat et al., 2020), suggesting that there may be other factors shaping the honeybee gut microbiome. By consulting the relevant literature, we found that there may also be differences in dietary habits among different honeybee species (DeGrandi-Hoffman et al., 2013; Urcan et al., 2018; Disayathanoowat et al., 2020). However, little is known about the interactions between dietary habits, especially dietary structure preference, and the colonization patterns of gut microbiota among different honeybee species.

Studies on stomach of mammals (Noto and Peek, 2017) and the midgut of some insects, such as mosquitoes (Boudko et al., 2001) and termites (Tim et al., 2012), have shown that although the gastrointestinal intragastric environment is harsh, there are also small but structured bacterial communities. Our recent study found that the emptying time of food in the midgut of western honeybees is within 12–24h (Wang et al., 2020c). The persistent peritrophic membrane structure in the midgut provides a good medium for microbial attachment and colonization. Although previous works have shown that the number of microorganisms in the midgut of honeybees is small (Disayathanoowat et al., 2020), it is worth further exploring that whether bees have the same pattern characteristics as other insects or mammals.

The purposes of this study were to investigate the effects of dietary habits on the shape of honeybee gut microbial communities. To address this issue, we first analyzed the main differences in the dietary structure of the two species of sympatric honeybees and provided evidence that their dietary habits were different. Then, we compared the differences in intestinal microbial community composition and growth and development before and after the change in dietary habits of different bee species. The results indicate that the different midgut microbiota community compositions between the two bee species may be shaped by their native dietary habits. Moreover, we found that changes in dietary habits have a negative effect on the development of honeybee midguts and hypopharyngeal glands (HPGs).

## Materials and Methods

### Bee and Bee Bread Sampling

Three colonies each of *Apis mellifera ligustica* (*Apis mellifera*) and *Apis cerana* (*Apis cerana*) were used in this study. All honeybee colonies were kept in the same apiary located at Shandong Agricultural University in Tai’an, Shandong Province, China. In the early spring, combs with stored bee bread were all replaced with empty combs, and all of the bee and bee bread samples were collected in May 2018.

**Figure 1:**
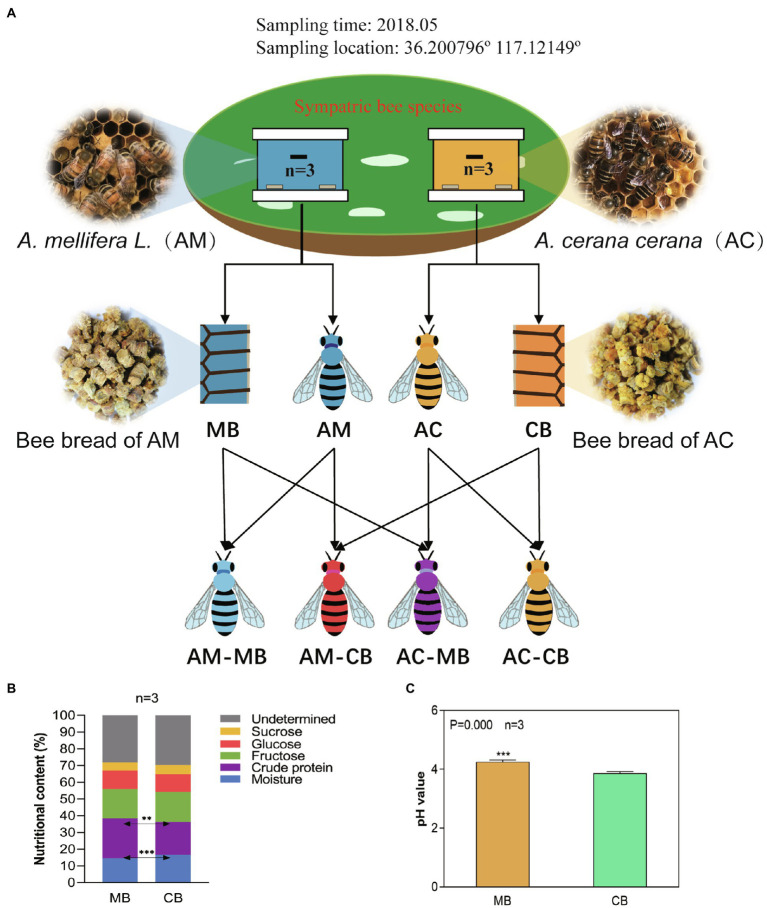
Experimental design **(A)** and the comparison of the chemical composition **(B)** and pH **(C)** of bee bread made by *Apis cerana* and *Apis mellifera*. **(A)** Experimental design: Honeybee and bee bread samples were obtained from sympatric *Apis mellifera* and *Apis cerana* colonies (three colonies of each group). Honeybee samples of *Apis mellifera* bees fed *Apis mellifera* bee bread (AM-MB) and *Apis cerana* bees fed *Apis cerana* bee bread (AC-CB) were obtained from their natural colonies, and honeybee samples of *Apis mellifera* bees fed *Apis cerana* bee bread (AM-CB) and *Apis cerana* bees fed *Apis mellifera* bee bread (AC-MB) were obtained under laboratory-fed sterile conditions (methods). **(B)** Chemical compositions of bee bread made from *Apis cerana* and *Apis mellifera* (%, fresh weight). Data are reported as the mean of each group (Supplementary Figure S1). **(C)** Comparison of the pH of bee bread made by *Apis cerana* and *Apis mellifera*. Data are reported as the mean±SE. Statistical analysis was performed by independent samples *t*-test, ^**^p<0.01, and ^***^p<0.001.

The experimental design is shown in Figure 1A. For each colony, the following processes were conducted: 300g of hive-stored bee bread made by *Apis mellifera* and *Apis cerana* bees (grouped as MB and CB, respectively) was collected; newly emerged workers were color marked on the abdomen, and 50 of those were removed from each colony 9days after emergence (grouped as AM-MB in *Apis mellifera* and AC-CB in *Apis cerana*, respectively). To verify the effect of bee bread sources on midgut microorganisms, 50 newly emerged workers were collected from each colony and maintained in sterilized cages in a sterilized incubator (30°C, 55% RH; Wang et al., 2014). To avoid the influence of external microorganisms on these workers, we selected only upcoming bees with complete wax caps using a tag tracking method by Wang et al. (2015). The *Apis mellifera* bees were fed CB (AM-CB), the *Apis cerana* bees were fed MB (AC-MB), and 10 of those bees were removed from each cage 9days after emergence. We stress that during the experimental period, the caged AM-CB and AC-MB bees were supplied with plenty of sterilized water and sterilized sugar water (50% sucrose, w/v).

### Physical and Chemical Analyses

Bee bread (0.5g) or 10 midguts were used for the pH value test. The samples were dissolved in distilled water (1:1) in centrifuge tubes, homogenized, and centrifuged at 10,000×*g* for 5min. The pH values of the supernatants were measured using a pH meter (AS-pH-11, AS ONE, Osaka, Japan).

The moisture content of the bee bread samples was measured at 105°C until a constant weight was achieved. The analyses of the crude protein, fructose, glucose, and sucrose contents and a midgut development index were determined using the methods reported by previous studies (Wang et al., 2014, 2016).

To assess digestive enzyme activities, 120 midguts from the four groups with three repetitions were used to determine the digestive enzyme activities. Sample preparation was performed as described by Zhang et al. (2015a). To avoid the influence of food factors on midgut enzyme activity and digestive physiology, midgut samples without chyme were excluded from sampling. The digestive enzyme activities of midgut proteinase, amylase, invertase, and lipase were assayed with the corresponding commercial enzyme activity assay kits (Jiancheng Institute of Biological Engineering, Nanjing, China), and the test methods were carried out according to the instructions.

To estimate the developmental status of the midgut, 10 midguts of 9-day-old workers from each group were separated and rapidly transferred to a 10% buffered neutral formaldehyde solution (pH 7.2). The midgut thickness of the collected bees was measured using the methods reported by Wang et al. (2014).

For HPG morphometric measurements, HPGs of 15 bees from each group with three repetitions were dissected immediately using a binocular stereomicroscope (SMZ18, Nikon, Tokyo, Japan). Follow-up procedures were carried out according to the method reported by Jianke et al. (2010).

### DNA Extraction and PCR Amplification

For DNA extraction, 0.5g bee bread or 10 midguts of each sample from each group (*n*=3, three repetitions samples) were added to sterile centrifuge tubes and stored at −80°C until use. All further DNA extraction and PCR amplification steps were performed as previously described (Guo et al., 2017).

Amplicons were extracted from 2% agarose gels and purified using the AxyPrep DNA Gel Extraction Kit (Axygen Biosciences, Union City, CA, United States) according to the manufacturer’s instructions and quantified using QuantiFluor-ST (Promega, United States). Purified amplicons were pooled in equimolar ratios and paired-end sequenced (2×250) on an Illumina platform by NovaSeq 6,000 according to standard protocols. The raw reads were deposited into the NCBI Sequence Read Archive database (BioProject ID: PRJNA695151).

### Bioinformatics Analysis

To ensure the statistical reliability and biological validity of the subsequent analysis, the raw data were pretreated using previously described methods (Wang et al., 2019). In brief, paired-end clean reads were merged as raw tags using FLASH (Magoč and Salzberg, 2011; version 1.2.11). Noisy raw tag sequences were filtered by QIIME (Caporaso et al., 2010; version 1.9.1) under specific filtering conditions (Caporaso et al., 2010) to obtain high-quality clean tags. The clean tags were compared to the reference database^1^ using the UCHIME algorithm^2^ to detect chimeric sequences. All chimeric tags were removed to obtain effective tags for further analysis.

UPARSE (Edgar, 2013) was used to cluster the effective tags of all samples into operational taxonomic units (OTUs) at an identity threshold of 97% similarity. The representative sequences were classified as organisms by a naive Bayesian model using an RDP classifier (Wang et al., 2007; version 2.2) that was based on the SILVA database (Version 132; Pruesse et al., 2007). The abundance of each taxonomic group was determined with a Perl script and visualized using R software (version 2.15.3). Because the obtained sequences, especially the bee food treatment group, contained a large number of chloroplast and mitochondrial sequences, we filtered these sequences before analysis. The stacked bar plot of the community composition was visualized in the R project ggplot2 package (version 2.2.1; Wickham, 2016). All analyses of alpha and beta diversity indexes were performed with QIIME (Caporaso et al., 2010), and R software (version 2.15.3) was used to analyze beta diversity index differences among groups. To compare the alpha indexes among groups, Tukey’s HSD test was performed in SPSS 21.0 software (SPSS Inc., Chicago, Illinois). Multivariate statistical analyses, including a principal coordinate analysis (PCoA) of unweighted UniFrac distances, were performed and plotted in R.

### Statistical Analysis

SPSS 21.0 software (SPSS Inc., Chicago, Illinois) was used for statistical analyses. The data with a normal distribution are expressed as the mean±SEM. A *t*-test was used for comparisons between two groups, and ANOVA with Tukey’s HSD *post hoc* test was performed for comparisons among multiple groups. For statistical data with a non-normal distribution, the Mann–Whitney U test was used for comparisons between two groups, and the Kruskal–Wallis H test was used for comparisons among multiple groups. Pearson’s correlation analysis was used to test the relationship between the two variables. Statistically significant differences were recognized at *p*<0.05.

## Results

### The Native Dietary Habits of *Apis cerana* Are Different From Those of *Apis mellifera*

To evaluate the differences in dietary preferences between the two honeybee species, we tested the five main nutritive components of *Apis cerana* bee bread (CB) and *Apis mellifera* bee bread (MB), including moisture, crude protein, fructose, glucose, and sucrose (Figure 1B; Supplementary Figure S1). Specifically, the results showed that the bee bread of *Apis cerana* contained significantly higher moisture and lower crude protein levels than that of *Apis mellifera* (*t*-test, *p*<0.001 and *p*<0.01, respectively). To ascertain whether the higher moisture and lower protein in the *Apis cerana* bee bread are a peculiarity of the forage available in this location, or whether this generally holds true across the species, we collected five groups of *Apis cerana* and *Apis mellifera* bee bread from Ji’ning City, Shandong Province, China, in August 2021 and measured their water and protein contents. The results were similar to those of samples collected from Tai’an (Supplementary Figure S2), indicating that the nutritional components of bee food brewed by the two species do have species specificity. Thus, the feeding habits of different honeybees may have interspecific specificity. No statistically significant difference was found between CB and MB in the contents of fructose, glucose, or sucrose (*t*-test, all *p*>0.05). To further verify the taste preference differences between *Apis cerana* and *Apis mellifera*, the acidity of CB and MB samples was tested (Figure 1C). The results indicated that the pH of bee bread made by *Apis mellifera* (4.22±0.18) was significantly higher than that of bee bread made by *Apis cerana* (3.86±0.17, *t*-test, *p*=0.000).

### Composition of Microbiomes in Bee Bread and Honeybee Midgut

The V3+V4 region of the 16S rRNA gene of 18 samples was sequenced on the Illumina NovaSeq 6,000 platform. After preliminary filtration, 78,001–192,780 effective sequences (Supplementary Table S1) were obtained. The sequences were further clustered into 1,402 OTUs based on 100% identity (Supplementary Table S2). The rarefaction curves showed that the sequence could represent the vast majority of microbial diversity in each sample (Supplementary Figure S3) with coverage of more than 99% (Supplementary Table S3), indicating that Illumina HiSeq sequencing was deep enough to represent all bacterial communities detected.

To clarify the characteristics of the native dietary habit and midgut microflora between the two bee species, we performed alpha diversity analyses using Tukey HSD tests. Under natural conditions, the bacterial species richness (Chao1) and diversity (Shannon) in bee bread and the midgut of *Apis mellifera* were similar to those of *Apis cerana*, which share a relatively common environment (Tukey HSD, all *p*>0.05; Supplementary Table S4). For *Apis mellifera*, no statistically significant difference was found between bee bread and midgut bacterial species richness by the Chao1 and Shannon indexes (Tukey HSD, all *p*>0.05; Supplementary Table S4). When the dietary habits changed, the midgut microbial community diversity of both *Apis mellifera* (AM-CB) and *Apis cerana* (AC-MB) bees was significantly increased (Supplementary Table S4). This was also true for the Ace and observed OTU indexes, which displayed similar patterns (Supplementary Table S4).

**Figure 2:**
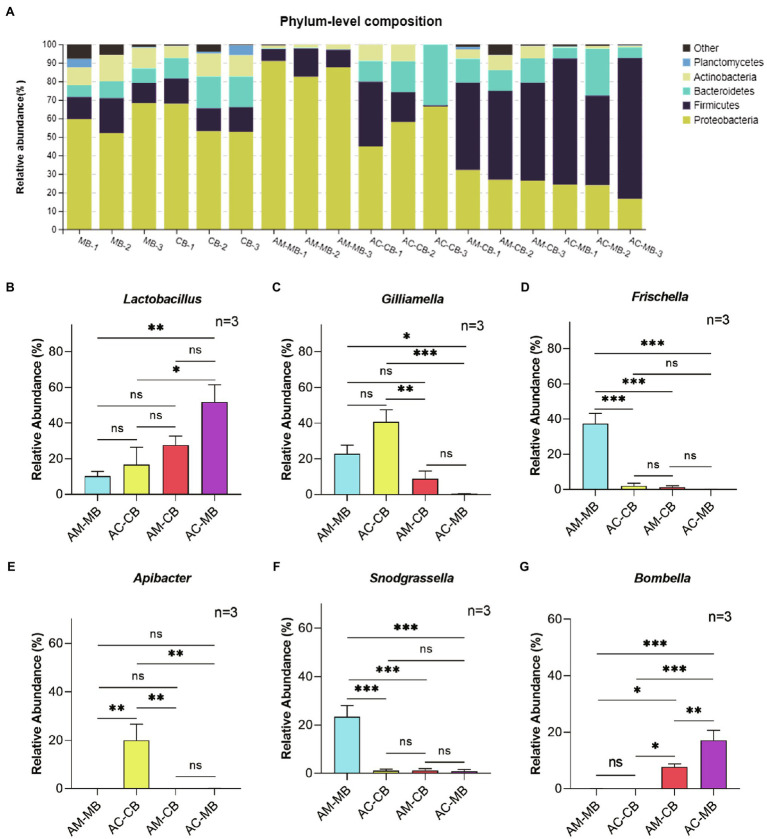
Comparison of major phyla and genera. **(A)** The relative abundances of the five most abundant bacterial phyla in all samples. **(B–G)** Comparison of the relative abundances of the genera Lactobacillus **(B)**, Gilliamella **(C)**, Frischella **(D)**, Apibacter **(E)**, Snodgrassella **(F)**, and Bombella **(G)** in the studied honeybee midgut samples. MB: Bee bread samples made by *Apis mellifera*. CB: Bee bread samples made by *Apis cerana*. AM-MB: Midgut samples of *Apis mellifera* bees fed *Apis mellifera* bee bread. AC-CB: Midgut samples of *Apis cerana* bees fed *Apis cerana* bee bread. AM-CB: Midgut samples of *Apis mellifera* bees fed *Apis cerana* bee bread. AC-MB: Midgut samples of *Apis cerana* bees fed *Apis mellifera* bee bread. Values are means±SME (*n*=3). Detailed results are reported in Supplementary Table S6. ^*^p<0.05, ^**^p<0.01, and ^***^p<0.001, by Tukey HSD test.

To clarify the microbial compositions of bee bread and midgut microflora, we assessed the relative abundances of the five most abundant bacterial phyla and five most abundant genera. In bee bread, the five most abundant bacterial phyla were Proteobacteria (57.88–59.97%), Firmicutes (13.13–13.92%), Bacteroidetes (7.79–14.95%), Actinobacteria (11.62–10.10%), and Planctomycetes (1.66–2.12%; Figure 2A; Supplementary Table S5). For natural bees, Proteobacteria (56.43–86.99%) was the most abundant bacterial phylum in the midgut, followed by Firmicutes (10.44–17.22%), Bacteroidetes (0.31–20.25%), Actinobacteria (1.97–6.02%), and Planctomycetes (less than 0.02%; Figure 2A; Supplementary Table S5). Compared with *Apis cerana* bees, a significantly higher abundance of Proteobacteria and a lower abundance of Bacteroidetes were found in the *Apis mellifera* bee midgut. For the diet intervention groups, the midgut microflora composition of both AM-CB and AC-MB was significantly changed (Figure 2A; Supplementary Table S5). Specifically, a significant increase in the phylum Firmicutes and a decrease in the phylum Proteobacteria were observed in these two groups compared to the corresponding species of natural workers (Figure 2A; Supplementary Table S5).

At the genus level, the relative abundances of *Lactobacillus*, *Gilliamella*, *Frischella*, *Snodgrassella*, and *Apibacter* in bee bread made by *Apis mellifera* were similar to those in bee bread made by *Apis cerana* (all *p*>0.05; Supplementary Table S6). For natural bees, *Lactobacillus*, *Gilliamella*, *Frischella*, *Snodgrassella*, and *Apibacter* were the five most abundant genera in the midgut of *Apis mellifera* bees and *Apis cerana* bees (Figures 2B–F; Supplementary Table S6). However, there were significant differences in the proportion of different kinds of bacteria between the two bee species. Compared with *Apis cerana* bees, a significantly higher abundance of *Frischella* and *Snodgrassella* and a lower abundance of *Apibacter* were found in the *Apis mellifera* bee midgut (Tukey HSD, all *p*<0.05; Figures 2B–F; Supplementary Table S6). When dietary patterns were changed, the unique characteristics of bacterial genera in the midgut between *Apis mellifera* and *Apis cerana* bees disappeared. More specifically, we found that the relative abundance of *Apibacter* in the midgut of AC-MB was significantly reduced (Tukey HSD, *p*<0.05), and the relative abundances of *Frischella* and *Snodgrassella* in the midgut of AM-CB were also significantly reduced (Tukey HSD, *p*<0.05; Figures 2B–F; Supplementary Table S6). Furthermore, the genus *Bombella*, which remained at extremely low levels in the midgut of natural *Apis mellifera* and *Apis cerana* bees, was more abundant in the AM-CB and AC-MB samples (Tukey HSD, all *p*<0.05; Figure 2G; Supplementary Table S6).

**Figure 3:**
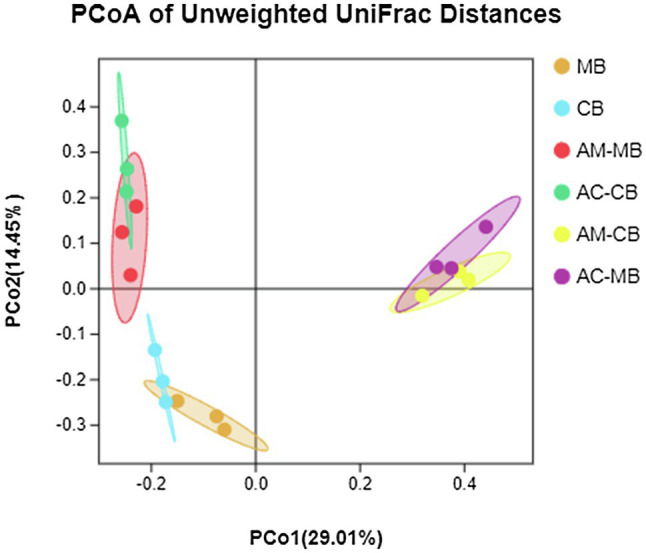
Principal coordinate analysis (PCoA) of unweighted UniFrac distances for all samples of the bee bread and midgut of *Apis mellifera* and *Apis cerana*. MB: Bee bread samples made by *Apis mellifera*. CB: Bee bread samples made by *Apis cerana*. AM-MB: Midgut samples of *Apis mellifera* bees fed *Apis mellifera* bee bread. AC-CB: Midgut samples of *Apis cerana* bees fed *Apis cerana* bee bread. AM-CB: Midgut samples of *Apis mellifera* bees fed *Apis cerana* bee bread. AC-MB: Midgut samples of *Apis cerana* bees fed *Apis mellifera* bee bread.

To evaluate beta diversity, a PCoA was performed using the unweighted UniFrac distances among groups. The results showed that the food microbial community composition was different from that of the midgut (Figure 3). The permutational MANOVA (PERMANOVA) results showed that there was no significant difference in the microbiota at the dietary pattern and species levels (Supplementary Table S7). However, the interactive effects of dietary patterns and species significantly altered the bee midgut microbiota compositions (Figure 3; Supplementary Table S7). Specifically, the interactive effects of dietary intervention significantly altered the bee midgut microbiota compositions more than normal dietary patterns or bee species alone (Figure 3; Supplementary Table S7).

To further evaluate the correlations between dietary habits and midgut bacteria of the two bee species, Pearson’s correlation analysis was performed between the three characteristic indexes of dietary habits and the five most abundant bacteria at the genus level. The results showed that the dietary patterns were significantly correlated with the relative abundance of midgut microbes (Supplementary Figures S4–S6; Supplementary Table S8). With the increase in the protein content, moisture content, and pH of bee bread, the dominant midgut microbes of *Apis mellifera* and *Apis cerana* showed opposite trends (Supplementary Figures S4–S6; Supplementary Table S8). However, the characteristic indexes of dietary type had different correlation coefficients with the relative abundances of midgut microbes (Supplementary Figures S4–S6; Supplementary Table S8). Here, we mainly focused on the dietary factors that were significantly associated with the characteristic midgut microbes of the two honeybee species. The results showed that the relative abundances of the genera *Frishella* and *Snodgrassella* in the midgut of *Apis mellifera* bees were positively correlated with the crude protein content and negatively correlated with the moisture content. The relative abundance of the genus *Apibacter* in the midgut of *Apis cerana* bees was negatively correlated with dietary pH and positively correlated with the moisture content (Supplementary Figures S4–S6; Supplementary Table S8).

### Effects of Changes in Dietary Habits on Digestive Physiology and Hypopharyngeal Gland Development in Honeybees

To further evaluate the effects of changes in dietary habits on digestive physiology and hypopharyngeal gland development in the two species of honeybees, we investigated the activity of digestive enzymes, the pH of the midgut, midgut thickness, and the development of the hypopharyngeal gland of 9-day-old workers under normal conditions or dietary intervention.

The variations in the activities of digestive enzymes, including proteinase, amylase, invertase, and lipase, are shown in Supplementary Figure S7. For *Apis mellifera*, except for the amylase activity of bees fed *Apis cerana* bee bread being significantly higher than that of natural bees (Turkey HSD, *p*<0.05), no statistically significant differences were observed in the activities of proteinase, invertase, or lipase (Turkey HSD, all *p*>0.05). For *Apis cerana*, a significant increase in invertase and amylase activities and a decrease in proteinase activity were observed in bees fed *Apis mellifera* bee bread compared to normal bees (Turkey HSD, all *p*<0.05). Furthermore, no statistically significant differences in the activity of lipase were observed between the two *Apis cerana* groups (Tukey HSD, *p*>0.05).

**Figure 4:**
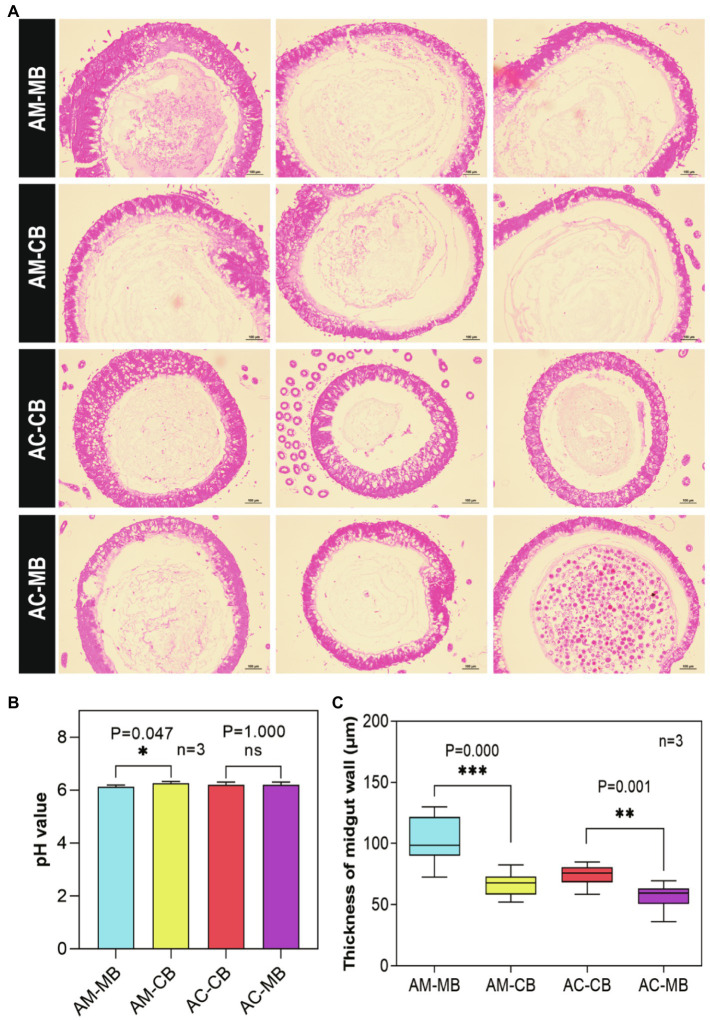
Effect of dietary habit changes on midgut development of 9-day-old *Apis mellifera* and *Apis cerana* workers under different treatments. **(A)** Cross section of the midgut of workers aged 9days old (H&E, 100×). **(B)** pH values in midguts of workers aged 9days old under different treatments. **(C)** Effects of dietary habit changes on midgut thickness of workers aged 9days old under different treatments. Values are means±SME. ^*^p<0.05, ^**^p<0.01, and ^***^p<0.001 by independent samples *t*-test.

Next, we examined the effects of dietary habit changes on the midgut development of workers under different treatments (Figure 4). From the midgut sections, an obviously developed peritrophic membrane structure could be seen in all treatment groups (Figure 4A). Furthermore, a significantly higher pH was found in the midgut of natural *Apis mellifera* bees fed *Apis cerana* bee bread than in other bees (Figure 4B). No significant difference was found in the pH value of the midgut between the two *Apis cerana* groups (AC-CB vs. AC-MB; Figure 4B). Moreover, the following statistical analysis of midgut thickness under different treatments showed that the natural bees (AM-MB and AC-CB) had better developed midguts than the corresponding species in the dietary intervention groups (AM-CB and AC-MB, respectively; Figure 4C).

**Figure 5:**
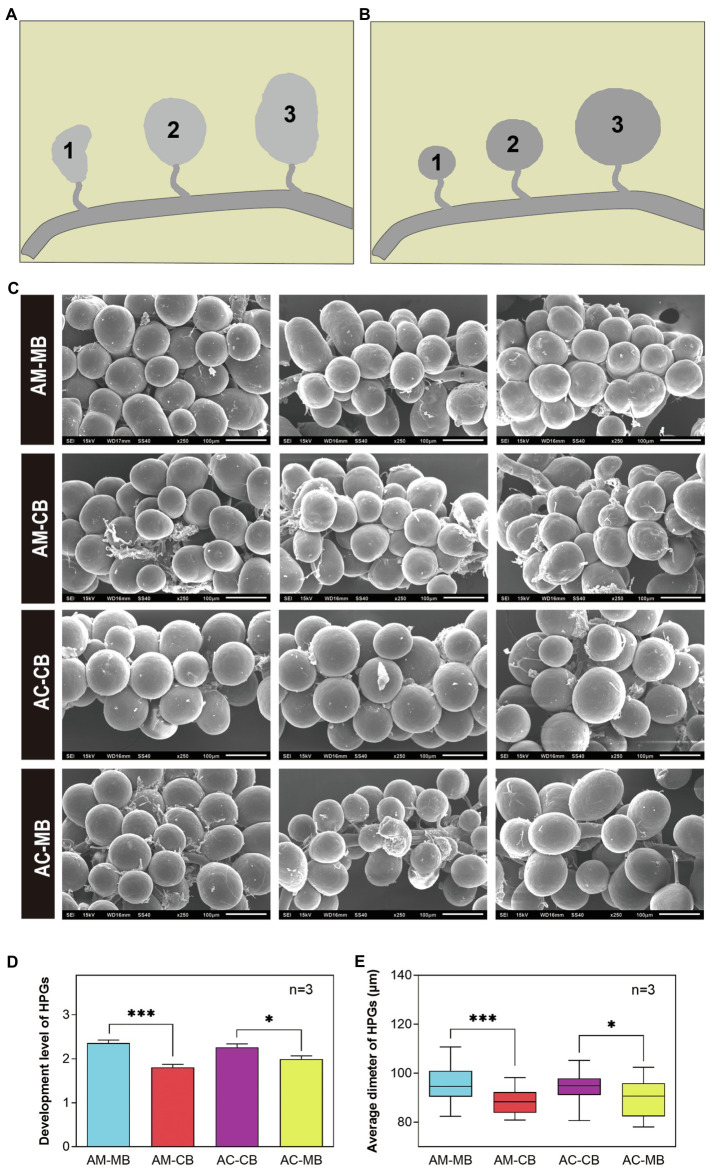
The morphology and development of hypopharyngeal glands (HPGs) among *Apis mellifera* and *Apis cerana* bees. **(A)** Morphology of HPGs under different treatments (SEM, ×250). **(B,C)** The development grading levels of *Apis mellifera* **(B)** and *Apis cerana* **(C)** bees, respectively. Level 1: The development of HPG is small and withered; Level 2: The development of HPG is moderately developed and plump; Level 3: The development of HPG is fully developed and plump. **(D,E)** Comparison of the development degree **(D)** and average sizes **(E)** of HPGs between *Apis mellifera* and *Apis cerana* bees. Values are means±SME. ^*^p<0.05, ^**^p<0.01, and ^***^p<0.001 by independent samples *t*-test.

Furthermore, we evaluated the developmental variations of hypopharyngeal glands between the four *Apis mellifera* and *Apis cerana* groups (Figure 5). Scanning electronic microscopy (SEM) photographs indicated that the shapes of hypopharyngeal glands were different between *Apis mellifera* and *Apis cerana* (Figure 5A). The hypopharyngeal gland of *Apis mellifera* was ellipsoid with a compact distribution, while a spherical shape with sparsely distributed glands was found in *Apis cerana*. It is obvious that the hypopharyngeal glands of natural bees (AM-MB and AC-CB) were plump with high uniformity, while less even and developed hypopharyngeal glands were commonly found in workers with dietary habit changes (AM-CB and AC-MB). To describe the development of hypopharyngeal glands quantitatively among treatments, we divided the morphology of the developed hypopharyngeal glands into three levels according to bee species traits and developmental degree (Figures 5B,C). The statistical results showed that both natural bees (AM-MB and AC-CB) had significantly more developed hypopharyngeal glands than workers with changed dietary habits (AM-CB and AC-MB, respectively; Figure 5D). This was also true for the acinar diameters of the hypopharyngeal glands, which displayed similar results (Figure 5E).

## Discussion

Similar to other animals, honeybees have simple and specific gut microbiota, making them good models for gut microbiota research (Zheng et al., 2018; Douglas, 2019). In this study, by focusing on the dietary habits and midgut microflora diversities of two sympatric honeybees, we sought to illuminate how dietary habits and host genetic background influence the gut microbiome under the same environmental conditions. Our results showed that the native dietary habit may play a key role in shaping the structural characteristics of bee midgut microbiota. Furthermore, we revealed a number of different features of the midgut microbiota between the two honeybee species that may be relevant to the host genetic background. We hope that these findings will provide valuable clues to better understand the relationships between diet, host, and intestinal microbiota.

### Dietary Habits Were Different Between *Apis mellifera* and *Apis cerana*

The differences in dietary habits among different races have been widely confirmed in many species, especially in humans (Filippo et al., 2010; Zhang et al., 2015b; Greene et al., 2020; Matoba et al., 2020; Wang et al., 2020b). Previous studies suggested that there may be significant differences in dietary habits among different honeybee species (DeGrandi-Hoffman et al., 2013; Disayathanoowat et al., 2020). In China, beekeepers discovered long ago that the collecting habits of *Apis cerana* were different from those of *Apis mellifera*. This phenomenon has recently been confirmed by Disayathanoowat et al. (2020), who found that *Apis mellifera* favors different main floral sources than *Apis cerana*, who shares a relatively common environment. In our study, we also found that the dietary structure of *Apis cerana* was significantly different from that of *Apis mellifera* (Figure 1B; Supplementary Figure S1), providing a stark indication that the native dietary habits of these two bee species were different. Pollen collected from different plant species varies considerably in chemical composition (Roulston et al., 2000), and the nutritional concentrations of bee bread depend on the plant sources, climatic conditions, and other environmental conditions (Baltrušaitytė et al., 2007; Urcan et al., 2017). Thus, the difference in dietary structure between *Apis cerana* and *Apis mellifera* in our study may be related to the differences in their collecting habits.

Taste is another important indicator of dietary habits. Previous studies suggested that different bee species have different preferences for bee bread acidity (DeGrandi-Hoffman et al., 2013, Disayathanoowat et al., 2020). Here, we also found that the pH of bee bread made by *Apis cerana* and *Apis mellifera* was significantly different (Figure 1C), but the pH value we measured was relatively lower than that in a previous study (5.82±0.02 for *Apis mellifera* and 5.90±0.02 for *Apis cerana*; Disayathanoowat et al., 2020). The inconsistent results may be related to the different plant sources and storage times under the two laboratory setups (Loper et al., 1980; Gilliam, 1997).

### Microbiome Similarities and Differences Between *Apis mellifera* and *Apis cerana*

Limited evidence of the interactions among pollen, bee food, and the bee gut system found that the α diversity of gut microbes in bees was highly similar to that of the bee bread and honey they ate (Anderson et al., 2013; Disayathanoowat et al., 2020). Our study showed that the microbial composition of bee breads made by sympatric *Apis mellifera* and *Apis cerana* honeybees was similar (Figure 2A; Supplementary Tables S3–S5), and this result was consistent with those of Disayathanoowat et al. (2020). Recent studies have shown that the environment first shapes the microorganisms of plants (Gong and Xin, 2021) and then shapes the microbiome of insects that depend on host plants for their food (Hannula et al., 2019). In this study, we found that there was no significant difference in the types of microorganisms between honeybee intestinal species and their food bee bread (Supplementary Tables S3–S5). The intestinal microbiomes of both *Apis mellifera* and *Apis cerana* were significantly changed under the dietary interventions (Figure 2; Supplementary Tables S3–S7). Since the bee bread made by *Apis mellifera* and *Apis cerana* has the same microbial composition, it seems that it was the diet compositions rather than dietary microorganisms that should be responsible for the change in midgut microbiome community composition. However, it is worth emphasizing that bee bread is not the only food-borne microorganism source for natural honeybees, and this may also be related to the physiological characteristics of the honeybee gut and the intestinal microorganisms carried by honeybees themselves.

Numerous studies have proven that adult workers have remarkably simple and conserved gut microbiota (Moran et al., 2012; Zheng et al., 2018; Ellegaard and Engel, 2019). However, it is generally thought that the midgut has few bacteria due to the existence of the peritrophic membrane structure (Engel and Moran, 2013). In this study, we confirmed that although the number of microorganisms in the midgut of sympatric natural *Apis mellifera* and *Apis cerana* honeybees was small and stable, the midgut was still relatively stable (Figure 2). The dominant microbiota established in the midguts of both bee species were Proteobacteria, Firmicutes, Bacteroidetes, Actinobacteria, and Planctomycetes, which is basically consistent with the results of previous studies on other honeybee intestinal segments (Ludvigsen et al., 2015; Kwong and Moran, 2016). Here, we must emphasize that, according to the limited research on the midgut microorganisms of bees, the midgut microbial composition of bees is easily affected by season, diet, age, and other factors (Ludvigsen et al., 2015; Kwong and Moran, 2016; Kešnerová et al., 2020; Ma et al., 2020; Wang et al., 2020a), which suggests that it is necessary to control environmental conditions in the study of honeybee intestinal microorganisms. Furthermore, our study reports for the first time that the core microflora in the midgut have strict host specificity between different honeybee species. This has further enriched the knowledge of bacterial communities within the honeybee gut.

**Figure 6:**
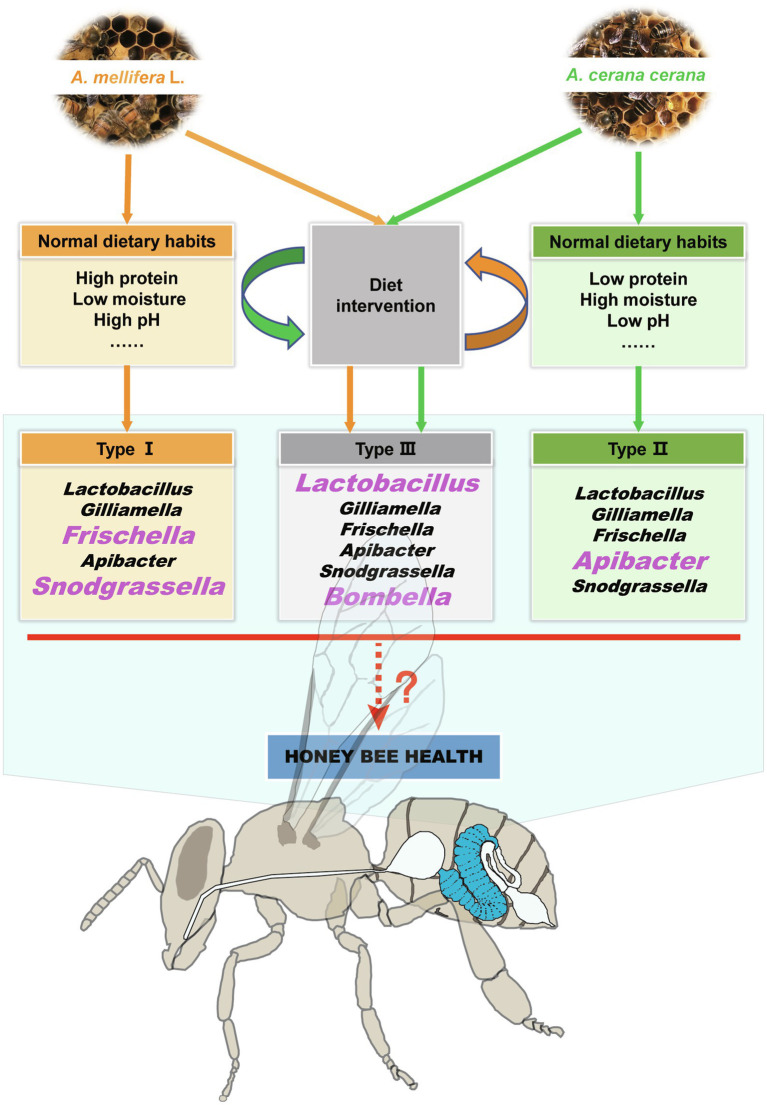
Summary of the effects of dietary habits on shaping the honeybee midgut microbiota and health. The sympatric *Apis mellifera* and *Apis cerana* bees have different dietary habits and midgut microbial characteristics. When their dietary habits changed, their unique microbiota signature disappeared, and a negative impact on the growth and development of the two kinds of honeybees was also confirmed. The bacterial genera in large pink font are the characteristic taxa in the corresponding host bee midguts.

Moreover, the gut microbiome is shaped by diet (Asnicar et al., 2021), and studies on humans and other animals have shown that changes in dietary habits or high dietary nutrient (e.g., protein, fat, and carbohydrate) intake could significantly change the structure of the intestinal microflora (Zhang et al., 2015b; Hussain et al., 2019; Han et al., 2020; Asnicar et al., 2021). In this study, our results suggested that there was a close linkage between the midgut microbiota and dietary nutrients and that dietary habit changes could significantly cause intestinal dysbacteriosis in honeybees (Supplementary Figures S3–S5; Supplementary Tables S8, S9). The specific manifestation of this effect is the disappearance of host-specific dominant microbiota (Figures 2, 6) and the increased relative abundance of *Lactobacillus* and some noncore bacteria (e.g., *Bombella*). Further studies on the relationship between dietary habits and gut microbiota will be helpful in understanding honeybee–microbiota interactions.

### Linkage Among Diet, Midgut Microbes, and Honeybee Development

It is well known that the midgut and hypopharyngeal gland development of honeybees (*Apis mellifera*) is significantly affected by the dietary protein concentration (DeGrandi-Hoffman et al., 2010; Wang et al., 2014; Omar et al., 2017). Previous studies have shown that there may be a close relationship between gut microbes and honeybee health (Barron, 2015; Goulson et al., 2015). Recently, a study on the changes in honeybee intestinal microbiota in different seasons found that gut bacterial loads were closely related to diet (Kešnerová et al., 2020). In our study, we also found that low-protein *Apis cerana* bee bread had negative effects on the growth and development of the *Apis mellifera* midgut and hypopharyngeal gland (Figures 4, 5, respectively). The gut microbiota play a critical role in honeybee growth (Zheng et al., 2017) and health (Raymann and Moran, 2018). Anecdotal evidence suggests that microbiota dysregulation is closely related to the occurrence of diseases and developmental disorders (Sherwin et al., 2018). Recently, scientists from Massachusetts General Hospital and other institutions have found that the gut microbiome is directly related to dietary habits, and the microbiome is also directly related to the levels of metabolic biomarkers of diseases (Asnicar et al., 2021). Different diets (e.g., high-fiber and high-protein diets) shape different gut microbial communities, which cause changes in metabolites by affecting gut microbial activity (Marzorati et al., 2017). Therefore, it is reasonable to believe that in this study, the imbalance of intestinal microbiota in workers caused by changes in dietary habits might be the main factor causing midgut and hypopharyngeal gland dysplasia.

Homeostasis of the midgut is essential to maintain the digestive and absorptive functions of honeybees. When the internal environment of the midgut changes due to diet, bees must take measures to keep the internal environment relatively stable, such as the relatively stable activities of digestive enzymes. This process is bound to mobilize and consume many nutrients in the body (especially the midgut), thus affecting the development of the midgut. This may explain why the high-protein *Apis mellifera* bee bread had negative effects on the growth and development of the *Apis cerana* midgut and hypopharyngeal gland (Figures 4, 5, respectively), while the digestive enzyme activities of the midgut did not appear to be affected (Supplementary Figure S7).

This study focuses on the native dietary habits of the two sympatric bee species and their effects on shaping midgut microorganisms. To test whether the differences in midgut microbiome structure between the two honeybees were caused by bee food, we set up a “diet conversion” experiment. Limited to the current research conditions, the experiment can only be tested by indoor feeding experiments. During the experiment, although, we tried our best to avoid the interference of external adverse factors, there were still some limitations in this study. First, we used bees form natural colonies as the control of cross-feeding experimental groups. As the intestinal microbiota composition of natural bees is jointly driven by dietary and other factors, the role of dietary habits in the shaping of bee intestinal flora may be amplified. Second, we are unable to assess whether the association results caused by dietary change are caused by dietary effect or cage effect, which would bias the results of our overall analyses toward the null hypothesis. Third, the study used a small sample size and was carried out in single and sympatric environment, which affects the validity and reliability of the experimental results to a certain extent. It would be very important to carry out experiments based on dietary habits data to further confirm our findings. Future studies in different regions will help to better understand how bee diets affect their physiology and gut communities. However, there are some challenges in assessing the effect of food type on gut microbiome composition. First, we lack more data to support the differences in dietary habits among different bee species. Second, studies on the interaction between dietary habits and intestinal microorganisms produce large and complex data sets, which is a great challenge for data analysis. Finally, to avoid results bias, a perfect experimental design and careful and standardized experimental control are necessary.

In summary, the results of this study demonstrated that specific dietary habits played an important role in shaping the honeybee midgut microbiome. Moreover, we revealed that changes in dietary habits will destroy the intestinal-specific microbiota composition, which is not conducive to the growth and development of honeybees. One possible mechanism is that the imbalance of intestinal microbiota caused by the change of dietary habits first affects the digestion and absorption function of the midgut and further affects the development of the hypopharyngeal gland (Figure 6). These findings will provide further understanding of the relationships among the environment, dietary habits, intestinal microorganisms, and bee health.

## Data Availability Statement

The datasets presented in this study can be found in online repositories. The names of the repository/repositories and accession number(s) can be found in the article/**Supplementary Material**.

## Author Contributions

YW and BX designed the study. ZLi, LM, and KH performed the experiments. GL and ZLiu contributed materials and analytical tools. YW and LM wrote the manuscript. GL and HW edited the manuscript. All authors contributed to the article and approved the submitted version.

## Funding

This work was financially supported by the National Natural Science Foundation (No. 31702195) and the Efficient Ecological Agriculture Innovation Project of the Taishan Industry Leading Talent Program (No. LJNY 202003).

## Conflict of Interest

The authors declare that the research was conducted in the absence of any commercial or financial relationships that could be construed as a potential conflict of interest.

## Publisher’s Note

All claims expressed in this article are solely those of the authors and do not necessarily represent those of their affiliated organizations, or those of the publisher, the editors and the reviewers. Any product that may be evaluated in this article, or claim that may be made by its manufacturer, is not guaranteed or endorsed by the publisher.

## Footnotes

^1^ http://drive5.com/uchime/uchime_download.html

^2^ http://www.drive5.com/usearch/manual/uchime_algo.html

## References

[ref1] Anderson K. E. Sheehan T. H. Mott B. M. Patrick M. Lucy S. Schwan M. R.. (2013). Microbial ecology of the hive and pollination landscape: bacterial associates from floral nectar, the alimentary tract and stored food of honeybees (*Apis mellifera*). PLoS One 8:e83125. doi: 10.1371/journal.pone.0083125, PMID: 24358254 PMC3866269

[ref2] Antonopoulos D. Huse S. M. Morrison H. G. Schmidt T. M. Sogin M. L. Young V. B. (2010). Reproducible community dynamics of the gastrointestinal microbiota following antibiotic perturbation. Infect. Immun. 77, 2367–2375. doi: 10.1128/IAI.01520-08, PMID: 19307217 PMC2687343

[ref3] Asnicar F. Berry S. E. Valdes A. M. Long H. N. Segata N. (2021). Microbiome connections with host metabolism and habitual diet from 1,098 deeply phenotyped individuals. Nat. Med. 2, 321–332. doi: 10.1038/s41591-020-01183-8, PMID: 33432175 PMC8353542

[ref4] Baltrušaitytė V. Venskutonis P. R. Čeksterytė V. (2007). Radical scavenging activity of different floral origin honey and beebread phenolic extracts. Food Chem. 101, 502–514. doi: 10.1016/j.foodchem.2006.02.007

[ref5] Barron A. B. (2015). Death of the bee hive: understanding the failure of an insect society. Curr. Opin. Insect Sci. 10, 45–50. doi: 10.1016/j.cois.2015.04.004, PMID: 29588013

[ref6] Bleha R. Shevtsova T. S. Kruík V. Korpilová T. Sinica A. (2019). Bee breads from two regions of eastern Ukraine: composition, physical properties and biological activities. Czech J. Food Sci. 37, 9–20. doi: 10.17221/201/2018-CJFS

[ref7] Boudko D. Y. Moroz L. L. Linser P. J. Trimarchi J. R. Harvey W. R. (2001). In situ analysis of ph gradients in mosquito larvae using non-invasive, self-referencing, pH-sensitive microelectrodes. J. Exp. Biol. 204, 691–699. doi: 10.1242/jeb.204.4.691, PMID: 11171351

[ref8] Burke C. Steinberg P. Rusch D. Kjelleberg S. Thomas T. (2011). Bacterial community assembly based on functional genes rather than species. Proc. Natl. Acad. Sci. U. S. A. 108, 14288–14293. doi: 10.1073/pnas.1101591108, PMID: 21825123 PMC3161577

[ref9] Capcarova M. Kalafova A. Schwarzova M. Schneidgenova M. Brindza J. (2019). Consumption of bee bread influences glycaemia and development of diabetes in obese spontaneous diabetic rats. Biologia 75, 705–711. doi: 10.2478/s11756-019-00337-5

[ref10] Caporaso J. Kuczynski J. Stombaugh J. Bittinger K. Bushman F. (2010). QIIME allows integration and analysis of high-throughput community sequencing data. Nat. Methods 7, 335–336. doi: 10.1038/nmeth.f.303, PMID: 20383131 PMC3156573

[ref11] Caporaso J. G. Lauber C. L. Costello E. K. Berg-Lyons D. Gonzalez A. Stombaugh J.. (2011). Moving pictures of the human microbiome. Genome Biol. 12:R50. doi: 10.1186/gb-2011-12-5-r50, PMID: 21624126 PMC3271711

[ref12] Chatelier E. L. Nielsen T. Qin J. J. Prifti E. Hildebrand F. Falony G.. (2013). Richness of human gut microbiome correlates with metabolic markers. Nature 500, 541–546. doi: 10.1038/nature12506, PMID: 23985870

[ref13] Clemente J. Ursell L. Parfrey L. W. Knight R. (2012). The impact of the gut microbiota on human health: an integrative view. Cell 148, 1258–1270. doi: 10.1016/j.cell.2012.01.035, PMID: 22424233 PMC5050011

[ref14] David L. A. Maurice C. F. Carmody R. N. Gootenberg D. B. Button J. E. Wolfe B. E.. (2014). Diet rapidly and reproducibly alters the human gut microbiome. Nature 505, 559–563. doi: 10.1038/nature12820, PMID: 24336217 PMC3957428

[ref15] DeGrandi-Hoffman G. Chen Y. Huang E. Huang M. H. (2010). The effect of diet on protein concentration, hypopharyngeal gland development and virus load in worker honeybees (*Apis mellifera* L.). J. Insect Physiol. 56, 1184–1191. doi: 10.1016/j.jinsphys.2010.03.017, PMID: 20346950

[ref16] DeGrandi-Hoffman G. Eckholm B. J. Huang M. H. (2013). A comparison of bee bread made by africanized and European honeybees (*Apis mellifera*) and its effects on hemolymph protein titers. Apidologie 44, 52–63. doi: 10.1007/s13592-012-0154-9

[ref17] Disayathanoowat T. Li H. Supapimon N. Suwannarach N. Lumyong S. Chantawannakul P.. (2020). Different dynamics of bacterial and fungal communities in hive-stored bee bread and their possible roles: a case study from two commercial honeybees in China. Microorganisms 8:264. doi: 10.3390/microorganisms8020264, PMID: 32075309 PMC7074699

[ref18] Douglas A. E. (2019). Simple animal models for microbiome research. Nat. Rev. Microbiol. 17, 764–775. doi: 10.1038/s41579-019-0242-1, PMID: 31417197

[ref19] Edgar R. C. (2013). UPARSE: highly accurate OTU sequences from microbial amplicon reads. Nat. Methods 10, 996–998. doi: 10.1038/nmeth.2604, PMID: 23955772

[ref20] Ellegaard K. M. Engel P. (2019). Genomic diversity landscape of the honeybee gut microbiota. Nat. Commun. 10:446. doi: 10.1038/s41467-019-08303-0, PMID: 30683856 PMC6347622

[ref21] Engel P. Kwong W. K. McFrederick Q. Anderson K. E. Barribeau S. M. Chandler J. A.. (2016). The bee microbiome: impact on bee health and model for evolution and ecology of host-microbe interactions. mBio 7, e02164–e02115. doi: 10.1128/mBio.02164-15, PMID: 27118586 PMC4850275

[ref22] Engel P. Moran N. A. (2013). The gut microbiota of insects-diversity in structure and function. FEMS Microbiol. Rev. 37, 699–735. doi: 10.1111/1574-6976.12025, PMID: 23692388

[ref23] Falony G. Joossens M. Vieira-Silva S. Wang J. Darzi Y. Faust K.. (2016). Population-level analysis of gut microbiome variation. Science 352, 560–564. doi: 10.1126/science.aad3503, PMID: 27126039

[ref24] Filippo C. D. Cavalieri D. Paola M. D. Ramazzotti M. Poullet J. B. Massart S.. (2010). Impact of diet in shaping gut microbiota revealed by a comparative study in children from Europe and rural Africa. Proc. Natl. Acad. Sci. U. S. A. 107, 14691–14696. doi: 10.1073/pnas.1005963107, PMID: 20679230 PMC2930426

[ref25] Gilliam M. (1997). Identification and roles of non-pathogenic microflora associated with honeybees. FEMS Microbiol. Lett. 155, 1–10. doi: 10.1016/S0378-1097(97)00337-6

[ref26] Goldford J. E. Lu N. X. Bajic D. Estrela S. Tikhonov M. Sanchez-Gorostiaga A.. (2018). Emergent simplicity in microbial community assembly. Science 361, 469–474. doi: 10.1126/science.aat1168, PMID: 30072533 PMC6405290

[ref27] Gong T. Xin X. F. (2021). Phyllosphere microbiota: community dynamics and its interaction with plant hosts. J. Integr. Plant Biol. 63, 297–304. doi: 10.1111/jipb.13060, PMID: 33369158

[ref28] Goulson D. Nicholls E. Botías C. Rotheray E. L. (2015). Bee declines driven by combined stress from parasites, pesticides, and lack of flowers. Science 347:1435. doi: 10.1126/science.1255957, PMID: 25721506

[ref29] Greene L. K. Williams C. V. Junge R. E. Mahefarisoa K. L. Rajaonarivelo T. Rakotondrainibe H.. (2020). A role for gut microbiota in host niche differentiation. ISME J. 14, 1675–1687. doi: 10.1038/s41396-020-0640-4, PMID: 32238913 PMC7305313

[ref30] Guo M. Wu F. Hao G. Qi Q. Li R. Li N.. (2017). *Bacillus subtilis* improves immunity and disease resistance in rabbits. Front. Immunol. 8:354. doi: 10.3389/fimmu.2017.00354, PMID: 28424690 PMC5372816

[ref31] Han M. Yang K. Yang P. Zhong C. Chen C. Wang S.. (2020). Stratification of athletes’ gut microbiota: the multifaceted hubs associated with dietary factors, physical characteristics and performance. Gut Microbes 12, 1–18. doi: 10.1080/19490976.2020.1842991, PMID: 33289609 PMC7734118

[ref32] Hannula S. E. Zhu F. Heinen R. Bezemer T. M. (2019). Foliar-feeding insects acquire microbiomes from the soil rather than the host plant. Nat. Commun. 10:1254. doi: 10.1038/s41467-019-09284-w, PMID: 30890706 PMC6425034

[ref33] Hussain M. Bonilla-Rosso G. Kwong Chung C. K. C. Bäriswyl L. Rodriguez M. P. Kim B. S.. (2019). High dietary fat intake induces a microbiota signature that promotes food allergy. J. Allergy Clin. Immunol. 144, 157.e8–170.e8. doi: 10.1016/j.jaci.2019.01.043, PMID: 30768991

[ref34] Jianke L. Mao F. Begna D. Yu F. Aijuan Z. (2010). Proteome comparison of hypopharyngeal gland development between Italian and royal jelly producing worker honeybees (*Apis mellifera* L.). J. Proteome Res. 9, 6578–6594. doi: 10.1021/pr100768t, PMID: 20882974

[ref35] Kartzinel T. R. Hsing J. C. Musili P. M. Brown B. R. P. Pringle R. M. (2019). Covariation of diet and gut microbiome in African megafauna. Proc. Natl. Acad. Sci. U. S. A. 116, 23588–23593. doi: 10.1073/pnas.1905666116, PMID: 31685619 PMC6876249

[ref36] Kešnerová L. Emery O. Troilo M. Liberti J. Erkosar B. Engel P. (2020). Gut microbiota structure differs between honeybees in winter and summer. ISME J. 14, 801–814. doi: 10.1038/s41396-019-0568-8, PMID: 31836840 PMC7031341

[ref37] Khalifa S. A. M. Elashal M. Kieliszek M. Ghazala N. E. Farag M. A. Saeed A.. (2020). Recent insights into chemical and pharmacological studies of bee bread. Trends Food Sci. Technol. 97, 300–316. doi: 10.1016/j.tifs.2019.08.021

[ref38] Kwong W. K. Medina L. A. Koch H. Sing K. W. Soh E. J. Y. Ascher J. S.. (2017). Dynamic microbiome evolution in social bees. Sci. Adv. 3:e1600513. doi: 10.1126/sciadv.1600513, PMID: 28435856 PMC5371421

[ref39] Kwong W. K. Moran N. A. (2016). Gut microbial communities of social bees. Nat. Rev. Microbiol. 14, 374–384. doi: 10.1038/nrmicro.2016.43, PMID: 27140688 PMC5648345

[ref40] Loper G. M. Standifer L. N. Thompson M. J. Gilliam M. (1980). Biochemistry and microbiology of bee-collected almond (*Prunus dulcis*) pollen and bee bread. I-fatty acids, sterols, vitamins and minerals. Apidologie 11, 63–73. doi: 10.1051/apido:19800108

[ref41] Ludvigsen J. Rangberg A. Avershina E. Sekelja M. Kreibich C. Amdam G.. (2015). Shifts in the midgut/pyloric microbiota composition within a honeybee apiary throughout a season. Microbes Environ. 30, 235–244. doi: 10.1264/jsme2.ME15019, PMID: 26330094 PMC4567562

[ref42] Ma W. H. Zheng X. Li L. Shen J. Gao Y. (2020). Changes in the gut microbiota of honeybees associated with jujube flower disease. Ecotoxicol. Environ. Saf. 198:110616. doi: 10.1016/j.ecoenv.2020.110616, PMID: 32334202

[ref43] Magoč T. Salzberg S. L. (2011). FLASH: fast length adjustment of short reads to improve genome assemblies. Bioinformatics 27, 2957–2963. doi: 10.1093/bioinformatics/btr507, PMID: 21903629 PMC3198573

[ref45] Martiny J. B. H. Jones S. E. Lennon J. T. Martiny A. C. (2015). Microbiomes in light of traits: a phylogenetic perspective. Science 350:aac9323. doi: 10.1126/science.aac9323, PMID: 26542581

[ref46] Marzorati M. Vilchez-Vargas R. Bussche J. V. Truchado P. Jauregui R. Hage R. E.. (2017). High-fiber and high-protein diets shape different gut microbial communities, which ecologically behave similarly under stress conditions, as shown in a gastrointestinal simulator. Mol. Nutr. Food Res. 61:1600150. doi: 10.1002/mnfr.201600150, PMID: 27374808

[ref47] Matoba N. Akiyama M. Ishigaki K. Kanai M. Takahashi A. Momozawa Y.. (2020). GWAS of 165,084 Japanese individuals identified nine loci associated with dietary habits. Nat. Hum. Behav. 4, 308–316. doi: 10.1038/s41562-019-0805-1, PMID: 31959922

[ref48] Mayda N. Ozkok A. Bayram N. E. Gercek Y. C. Sorkun K. (2020). Bee bread and bee pollen of different plant sources: determination of phenolic content, antioxidant activity, fatty acid and element profiles. J. Food Meas. Charact. 14, 1795–1809. doi: 10.1007/s11694-020-00427-y

[ref49] Moran N. A. Hansen A. K. Powell J. E. Sabree Z. L. (2012). Distinctive gut microbiota of honeybees assessed using deep sampling from individual worker bees. PLoS One 7:e36393. doi: 10.1371/journal.pone.0036393, PMID: 22558460 PMC3338667

[ref500] Noto J. M. Peek R. M. (2017). The gastric microbiome, its interaction with helicobacter pylori, and its potential role in the progression to stomach cancer. Plos Pathogens. 13:e1006573. doi: 10.1371/journal.ppat.1006573, PMID: 28982167 PMC5629027

[ref50] Oh J. Byrd A. L. Park M. Kong H. H. Segre J. A. Sequencing N. C. (2016). Temporal stability of the human skin microbiome. Cell 165, 854–866. doi: 10.1016/j.cell.2016.04.008, PMID: 27153496 PMC4860256

[ref51] Omar E. Abd-Ella A. A. Khodairy M. M. Moosbeckhofer R. Crailsheim K. Brodschneider R. (2017). Influence of different pollen diets on the development of hypopharyngeal glands and size of acid gland sacs in caged honeybees (*Apis mellifera*). Apidologie 48, 425–436. doi: 10.1007/s13592-016-0487-x

[ref52] Pasolli E. Filippis F. D. Mauriello I. E. Cumbo F. Ercolini D. (2020). Large-scale genome-wide analysis links lactic acid bacteria from food with the gut microbiome. Nat. Commun. 11:2610. doi: 10.1038/s41467-020-16438-8, PMID: 32451391 PMC7248083

[ref53] Pruesse E. Quast C. Knittel K. Fuchs B. M. Ludwig W. Peplies J.. (2007). SILVA: a comprehensive online resource for quality checked and aligned ribosomal RNA sequence data compatible with ARB. Nucleic Acids Res. 35, 7188–7196. doi: 10.1093/nar/gkm864, PMID: 17947321 PMC2175337

[ref54] Raymann K. Moran N. A. (2018). The role of the gut microbiome in health and disease of adult honeybee workers. Curr. Opin. Insect Sci. 26, 97–104. doi: 10.1016/j.cois.2018.02.012, PMID: 29764668 PMC6010230

[ref55] Rothschild D. Weissbrod O. Barkan E. Kurilshikov A. Korem T. Zeevi D.. (2018). Environment dominates over host genetics in shaping human gut microbiota. Nature 555, 210–215. doi: 10.1038/nature25973, PMID: 29489753

[ref56] Roulston T. H. Cane J. H. Buchmann S. L. (2000). What governs protein content of pollen: pollinator preferences, pollen-pistil interactions, or phylogeny? Ecol. Monogr. 70:617. doi: 10.2307/2657188

[ref57] Sherwin E. Dinan T. G. Cryan J. F. (2018). Recent developments in understanding the role of the gut microbiota in brain health and disease. Ann. N. Y. Acad. Sci. 1420, 5–25. doi: 10.1111/nyas.13416, PMID: 28768369

[ref58] Sobral F. Calhelha R. C. Barros L. Duenas M. Tomas A. Santos-Buelga C.. (2017). Flavonoid composition and antitumor activity of bee bread collected in Northeast Portugal. Molecules 22:248. doi: 10.3390/molecules22020248, PMID: 28178217 PMC6155664

[ref59] Tim K. Carsten D. Scheffrahn R. H. Andreas B. (2012). High-resolution analysis of gut environment and bacterial microbiota reveals functional compartmentation of the gut in wood-feeding higher termites (*Nasutitermes* spp.). Appl. Environ. Microbiol. 78, 4691–4701. doi: 10.1128/AEM.00683-12, PMID: 22544239 PMC3370480

[ref60] Urcan A. C. Criste A. D. Dezmirean D. S. Mărgăoan R. Caeiro A. Campos M. G. (2018). Similarity of data from bee bread with the same taxa collected in India and Romania. Molecules 23:2491. doi: 10.3390/molecules23102491, PMID: 30274204 PMC6222490

[ref61] Urcan A. C. Marghitas L. A. Dezmirean D. S. Bobis O. Bonta V. Muresan C. I.. (2017). Chemical composition and biological activities of beebread: review. B. U. A. Sci. Vet. Med. C. L. 74, 6–14. doi: 10.15835/buasvmcn-asb:12646

[ref62] Wang Q. Garrity G. M. Tiedje J. M. Cole J. R. (2007). Naive bayesian classifier for rapid assignment of rRNA sequences into the new bacterial taxonomy. Appl. Environ. Microbiol. 73, 5261–5267. doi: 10.1128/AEM.00062-07, PMID: 17586664 PMC1950982

[ref63] Wang H. F. Liu C. L. Liu Z. G. Wang Y. Ma L. T. Xu B. H. (2020a). The different dietary sugars modulate the composition of the gut microbiota in honeybee during overwintering. BMC Microbiol. 20:61. doi: 10.1186/s12866-020-01726-6, PMID: 32183692 PMC7076957

[ref64] Wang B. Ma M. P. Diao Q. Y. Tu Y. (2019). Saponin-induced shifts in the rumen microbiome and metabolome of young cattle. Front. Microbiol. 10:356. doi: 10.3389/fmicb.2019.00356, PMID: 30873143 PMC6403146

[ref65] Wang Y. Ma L. T. Hang X. B. Yang W. R. Liu F. Xu B. H. (2014). Digestion of protein of two pollen types in China by the honeybee (*Apis mellifera* L). Apidologie 45, 590–600. doi: 10.1007/s13592-014-0278-1

[ref66] Wang Y. Ma L. T. Xu B. H. (2015). Diversity in life history of queen and worker honeybees, *Apis mellifera* L. J. Asia Pac. Entomol. 18, 145–149. doi: 10.1016/j.aspen.2014.11.005

[ref67] Wang Y. Ma L. T. Zhang W. X. Cui X. P. Wang H. F. Xu B. H. (2016). Comparison of the nutrient composition of royal jelly and worker jelly of honeybees (*Apis mellifera*). Apidologie 47, 48–56. doi: 10.1007/s13592-015-0374-x

[ref700] Wang Y. Ma L. T. Liu Z. G. Wang H. F. Xu B. H. (2020c). Processing time of three kinds of bee pollen in the digestive tract of Apis mellifera L. Chinese Journal of Applied Entomology. 57, 1111–1119. doi: 10.7679/j.issn.2095-1353.2020.112, PMID: 32183692

[ref68] Wang Z. B. Pang Y. J. Liu J. Wang J. Xie Z. Huang T. (2020b). Association of healthy lifestyle with cognitive function among Chinese older adults. Eur. J. Clin. Nutr. 75, 325–334. doi: 10.1038/s41430-020-00785-2, PMID: 33116235

[ref69] Wickham H. (2016). ggplot2: Elegant Graphics for Data Analysis. New York: Springer-Verlag

[ref70] Zhang J. Guo Z. Xue Z. Sun Z. Zhang M. Wang L.. (2015b). A phylo-functional core of gut microbiota in healthy young Chinese cohorts across lifestyles, geography and ethnicities. ISME J. 9, 1979–1990. doi: 10.1038/ismej.2015.11, PMID: 25647347 PMC4542028

[ref71] Zhang G. Zhang W. X. Cui X. P. Xu B. H. (2015a). Zinc nutrition increases the antioxidant defenses of honeybees. Entomol. Exp. Appl. 156, 201–210. doi: 10.1111/eea.12342

[ref72] Zheng H. Powell J. E. Steele M. I. Dietrich C. Moran N. A. (2017). Honeybee gut microbiota promotes host weight gain via bacterial metabolism and hormonal signaling. Proc. Natl. Acad. Sci. U. S. A. 114, 4775–4780. doi: 10.1073/pnas.1701819114, PMID: 28420790 PMC5422775

[ref73] Zheng H. Steele M. I. Leonard S. P. Motta E. V. S. Moran N. A. (2018). Honeybees as models for gut microbiota research. Lab. Anim. 47, 317–325. doi: 10.1038/s41684-018-0173-x, PMID: 30353179 PMC6478020

[ref74] Zhernakova A. Kurilshikov A. Bonder M. J. Tigchelaar E. F. Schirmer M. Vatanen T.. (2016). Population-based metagenomics analysis reveals markers for gut microbiome composition and diversity. Science 352, 565–569. doi: 10.1126/science.aad3369, PMID: 27126040 PMC5240844

