# Supplementary material for: The Native Dietary Habits of the Two Sympatric Bee Species and Their Effects on Shaping Midgut Microorganisms
Source: Front Microbiol. 2021 Oct 7;12:738226. doi: 10.3389/fmicb.2021.738226 (PMC8529121; doi:10.3389/fmicb.2021.738226)
Supplement: Supplementary file 1 [file Data_Sheet_1.zip › Supplementary Information.DOCX]

**Supplementary Information**

**The native dietary habits of the two sympatric bee species and their effects on shaping midgut microorganisms**

Ying Wang^1^, Zhenfang Li^2^, Lanting Ma^3^, Guilin Li^4^, Kai Han^3^, Zhenguo Liu^3^, Hongfang Wang^3^, Baohua Xu^3*^

^1^ Department of Science and Technology, Shandong Agricultural University, Taian, Shandong 271018, PR China

^2^ College of Plant Protection, China Agricultural University, Beijing 100083, PR China

^3^ College of Animal Science and Technology, Shandong Agricultural University, Taian, Shandong 271018, PR China

^4^ College of Life Sciences, Qufu Normal University, Jining, Shandong 273165, PR China

*Corresponding author: E-mail address: bhxu@sdau.edu.cn

**Supplementary Information includes:**

Figures S1 to S6

Tables S1 to S3, S5, S7 and S8


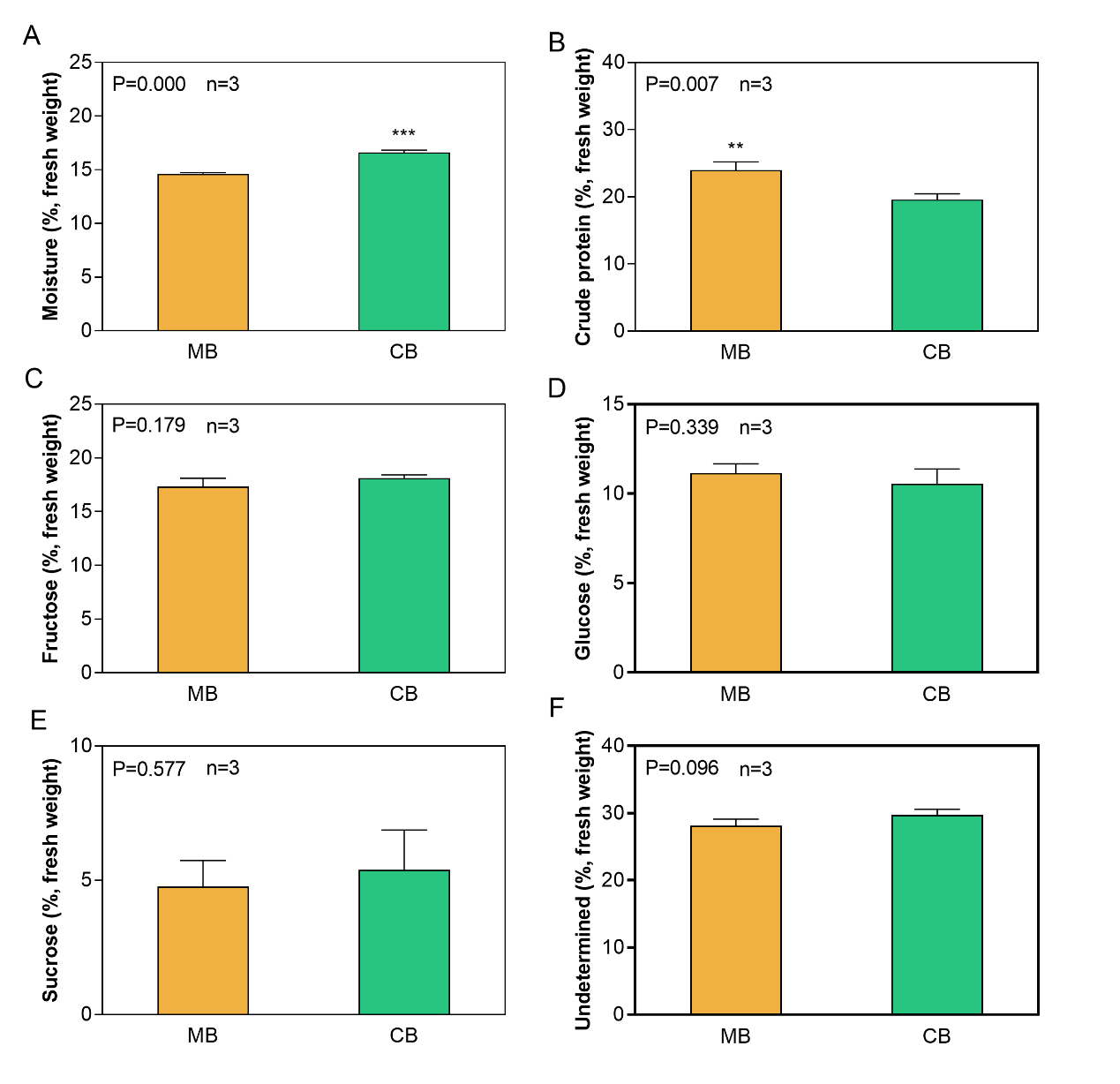


**Figure S1. Comparisons of moisture (A), crude protein (B), fructose (C), glucose (D), sucrose (E) and undetermined (F) contents in bee bread made by** ***Apis mellifera* and *Apis cerana*. MB**, *Apis mellifera* bee bread. **CB**: *Apis cerana* bee bread. Statistical analysis was performed by independent samples t-test, **P< 0.01, ***P< 0.001.


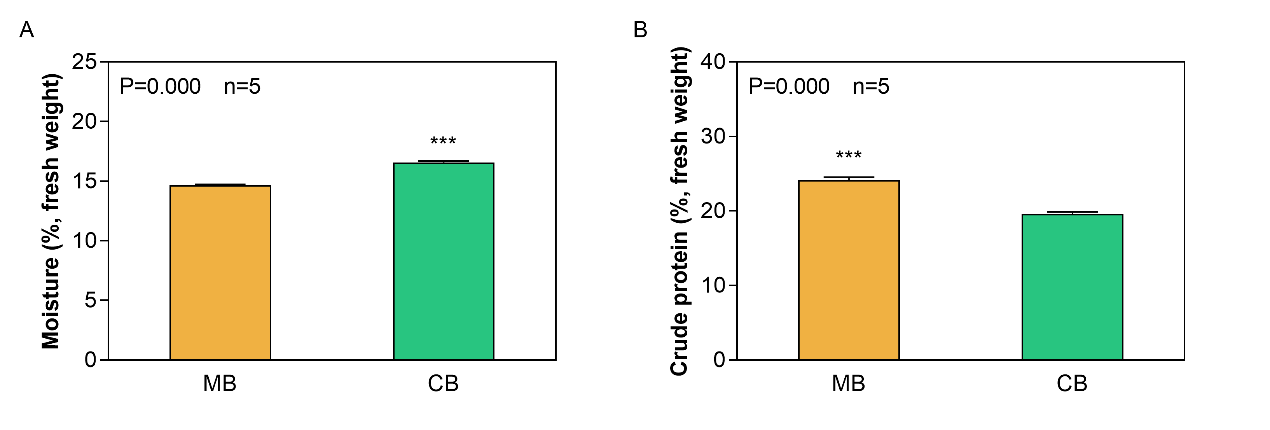


**Figure S2. Supplementary analysis of moisture (A) and crude protein (B) contents in bee bread made by** ***Apis mellifera* and *Apis cerana*. MB**, *Apis mellifera* bee bread. **CB**: *Apis cerana* bee bread. Statistical analysis was performed by independent samples t-test, ***P< 0.001. The bee bread samples were collected from 5 groups of *Apis cerana* and *Apis mellifera* bee bread from Ji'ning City, Shandong Province, China in August 2021.


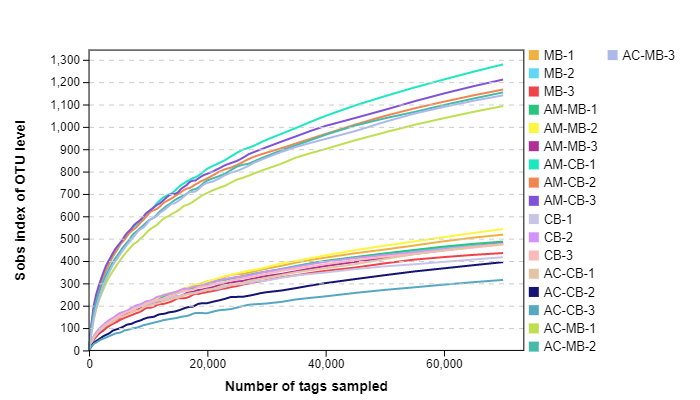


**Figure S3. The rarefaction analyses of all samples. Rarefaction curves generated from the OTUs suggested that high sampling coverage was achieved in all samples.** **MB (MB-1, MB-2, MB-3)**: Bee bread samples made by *Apis mellifera*. **CB (CB-1, CB-2, CB-3):** Bee bread samples made by *Apis cerana*. **AM-MB (AM-MB-1, AM-MB-2, AM-MB-3)**: Midgut samples of *Apis mellifera* bees fed *Apis mellifera* bee bread. **AC-CB (AC-CB-1, AC-CB-2, AC-CB-3)**: Midgut samples of *Apis cerana* bees fed *Apis cerana* bee bread. **AM-CB (AM-CB-1, AM-CB-2, AM-CB-3)**: Midgut samples of *Apis mellifera* bees fed *Apis cerana* bee bread. **AC-MB (AC-MB-1, AC-MB-2, AC-MB-3)**: Midgut samples of *Apis cerana* bees fed *Apis mellifera* bee bread.


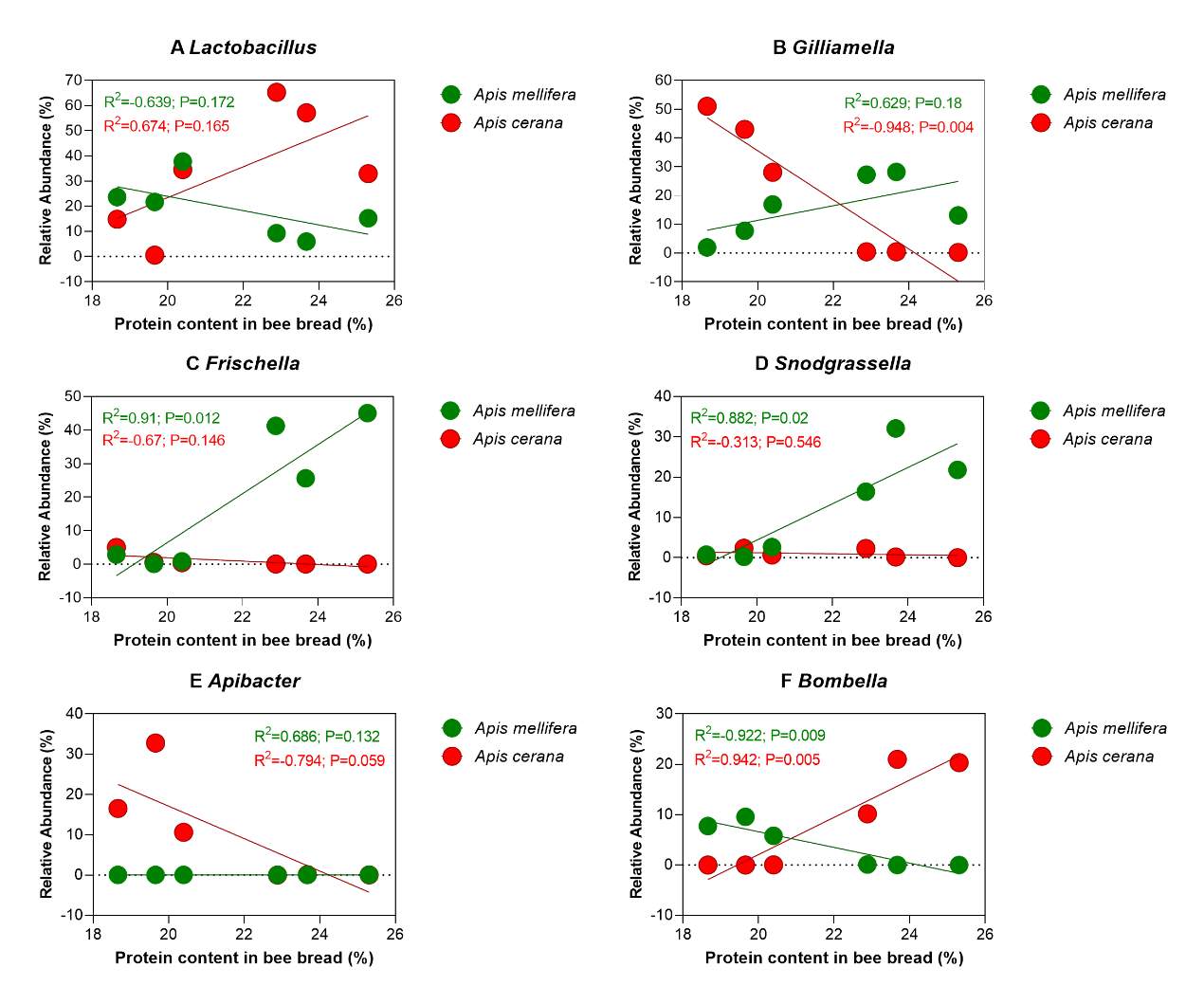


**Figure S4. Scatterplot of the dietary protein content and bacterial group proportion (genus level) fitted by a linear regression model. R^2^ and P values are shown in each figure and were the results of Pearson's correlation analysis.**


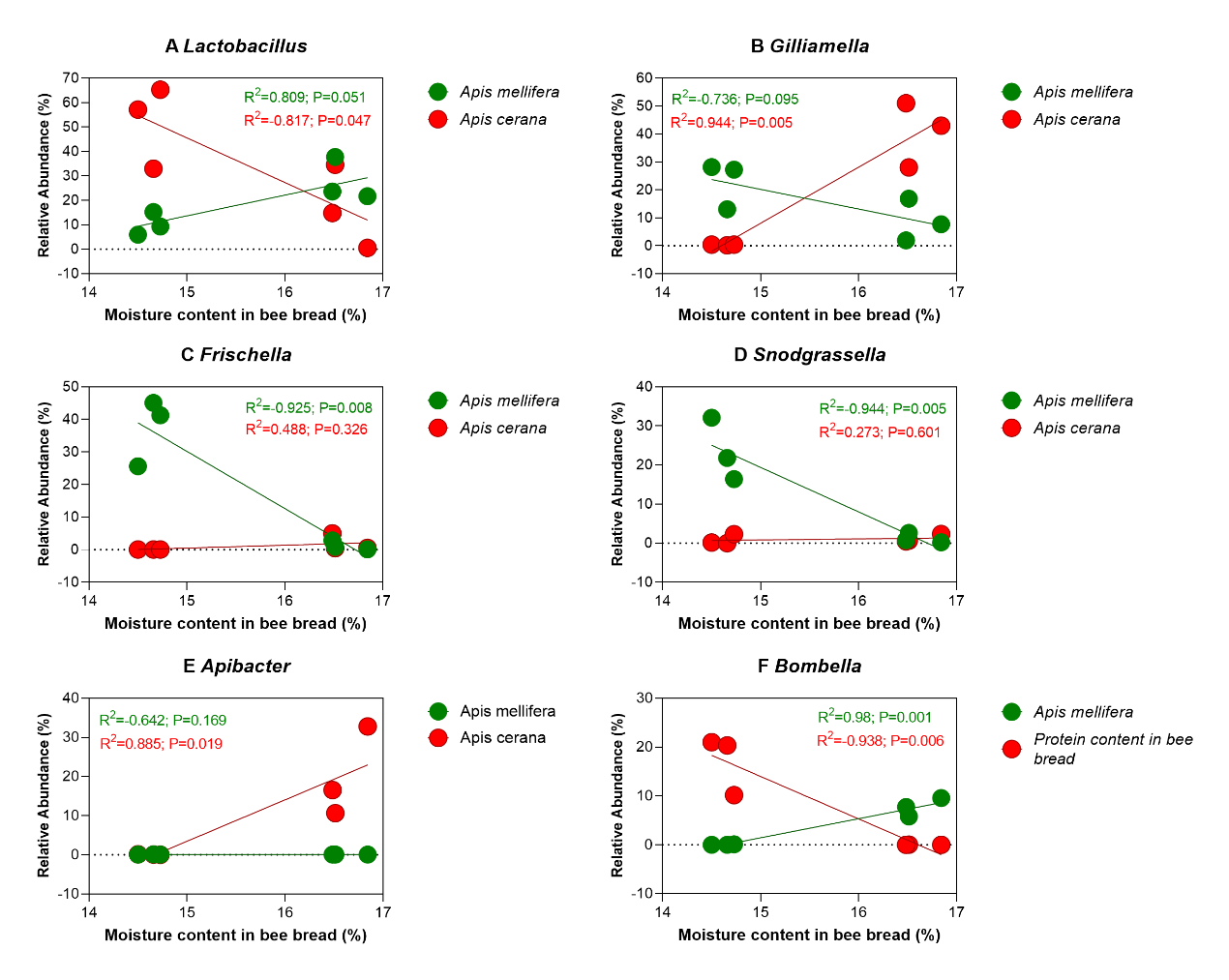


**Figure S5. Scatterplot of the dietary moisture content and bacterial group proportion (genus level) fitted by a linear regression model. R^2^ and P values are shown in each figure and were the results of Pearson's correlation analysis.**


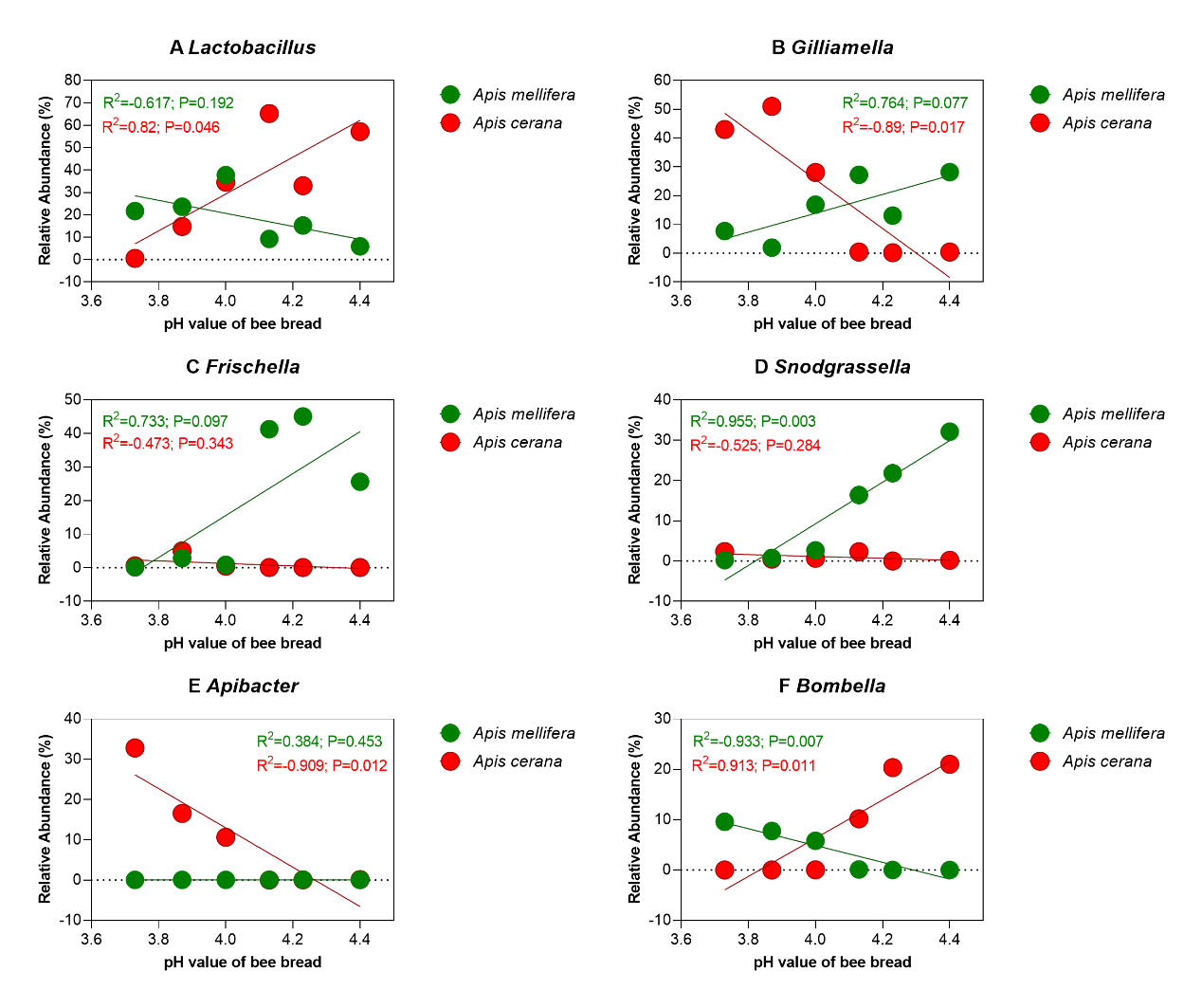


**Figure S6. Scatterplot of the dietary pH and bacterial group proportion (genus level) fitted by a linear regression model. R^2^ and P values are shown in each figure and were the results of Pearson's correlation analysis.**


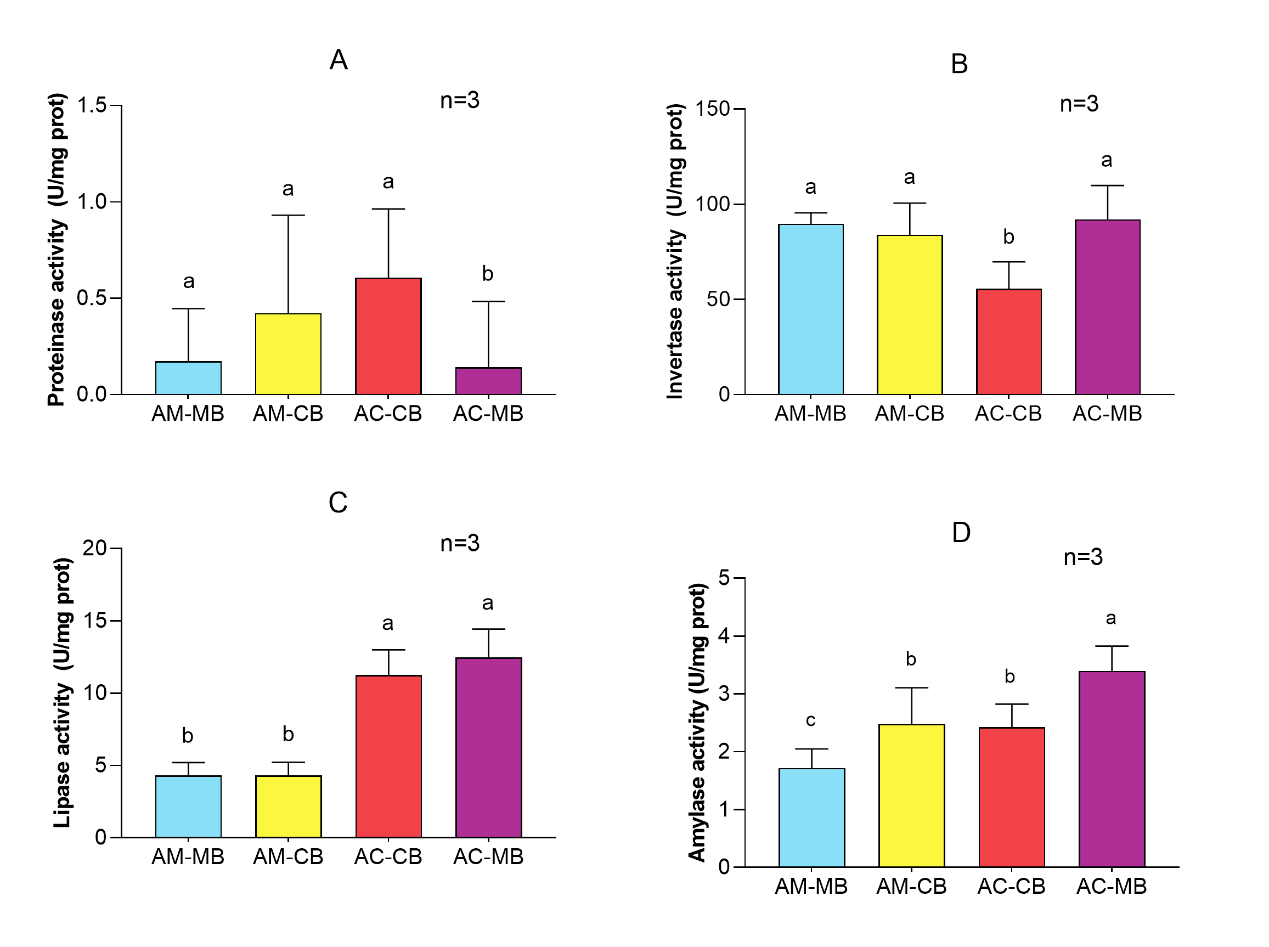


**Figure S7. Effect of dietary habit changes on proteinase, amylase, invertase, and lipase activites in the midgut of *Apis mellifera* and *Apis cerana*. MB**: Bee bread samples made by *Apis mellifera*. **CB**: Bee bread samples made by *Apis cerana*. **AM-MB**: Midgut samples of *Apis mellifera* bees fed *Apis mellifera* bee bread. **AC-CB**: Midgut samples of *Apis cerana* bees fed *Apis cerana* bee bread. **AM-CB**: Midgut samples of *Apis mellifera* bees fed *Apis cerana* bee bread. **AC-MB**: Midgut samples of *Apis cerana* bees fed *Apis mellifera* bee bread. Values are means ± SME. *P<0.05, **P<0.01 and ***P<0.001, by Tukey HSD test.

**Table S1. Statistical results of data preprocessing. MB (MB-1, MB-2, MB-3)**: Bee bread samples made by *Apis mellifera*. **CB (CB-1, CB-2, CB-3):** Bee bread samples made by *Apis cerana*. **AM-MB (AM-MB-1, AM-MB-2, AM-MB-3)**: Midgut samples of *Apis mellifera* bees fed *Apis mellifera* bee bread. **AC-CB (AC-CB-1, AC-CB-2, AC-CB-3)**: Midgut samples of *Apis cerana* bees fed *Apis cerana* bee bread. **AM-CB (AM-CB-1, AM-CB-2, AM-CB-3)**: Midgut samples of *Apis mellifera* bees fed *Apis cerana* bee bread. **AC-MB (AC-MB-1, AC-MB-2, AC-MB-3)**: Midgut samples of *Apis cerana* bees fed *Apis mellifera* bee bread.

| Sample Name | Raw PE | Clean PE | Raw Tags | Clean Tags | Effective Tags | Effective Ratio (%) |
| --- | --- | --- | --- | --- | --- | --- |
| AC-CB-1 | 100053 | 94848 | 94419 | 91710 | 91372 | 91.32 |
| AC-CB-2 | 117892 | 111505 | 111073 | 108161 | 107800 | 91.44 |
| AC-CB-3 | 114435 | 108030 | 107655 | 105059 | 104914 | 91.68 |
| AC-MB-1 | 92378 | 91995 | 90897 | 90342 | 88685 | 96 |
| AC-MB-2 | 100044 | 99634 | 98275 | 97542 | 95775 | 95.73 |
| AC-MB-3 | 98269 | 97846 | 96450 | 95791 | 93442 | 95.09 |
| AM-CB-1 | 103153 | 102583 | 100998 | 100144 | 98456 | 95.45 |
| AM-CB-2 | 90273 | 89831 | 88591 | 87660 | 86021 | 95.29 |
| AM-CB-3 | 99309 | 98767 | 97383 | 96439 | 94485 | 95.14 |
| AM-MB-1 | 124682 | 117367 | 116890 | 113752 | 113409 | 90.96 |
| AM-MB-2 | 117764 | 111116 | 108088 | 105552 | 105270 | 89.39 |
| AM-MB-3 | 84894 | 80437 | 80138 | 78202 | 78001 | 91.88 |
| CB-1 | 135845 | 128131 | 127664 | 125501 | 125339 | 92.27 |
| CB-2 | 115840 | 108622 | 108246 | 106269 | 106181 | 91.66 |
| CB-3 | 120620 | 113821 | 113442 | 111529 | 111444 | 92.39 |
| MB-1 | 125233 | 117596 | 116968 | 114185 | 114086 | 91.1 |
| MB-2 | 97293 | 92010 | 91404 | 89581 | 89524 | 92.01 |
| MB-3 | 211998 | 198565 | 197561 | 192987 | 192880 | 90.98 |

**Table S2. Statistics of OTUs and tags of different samples. MB (MB-1, MB-2, MB-3)**: Bee bread samples made by *Apis mellifera*. **CB (CB-1, CB-2, CB-3):** Bee bread samples made by *Apis cerana*. **AM-MB (AM-MB-1, AM-MB-2, AM-MB-3)**: Midgut samples of *Apis mellifera* bees fed *Apis mellifera* bee bread. **AC-CB (AC-CB-1, AC-CB-2, AC-CB-3)**: Midgut samples of *Apis cerana* bees fed *Apis cerana* bee bread. **AM-CB (AM-CB-1, AM-CB-2, AM-CB-3)**: Midgut samples of *Apis mellifera* bees fed *Apis cerana* bee bread. **AC-MB (AC-MB-1, AC-MB-2, AC-MB-3)**: Midgut samples of *Apis cerana* bees fed *Apis mellifera* bee bread.

| Sample ID | Total Tags | Unique Tags | Taxon Tags | Unclassified Tags | Singleton Tags | OTUs |
| --- | --- | --- | --- | --- | --- | --- |
| AM-CB-1 | 98456 | 64186 | 90878 | 0 | 7578 | 1402 |
| AM-CB-2 | 86021 | 52563 | 80917 | 0 | 5104 | 1224 |
| AM-CB-3 | 94485 | 61193 | 88136 | 0 | 6349 | 1313 |
| AM-MB-1 | 113409 | 49677 | 107771 | 0 | 5638 | 562 |
| AM-MB-2 | 105270 | 46301 | 100194 | 0 | 5076 | 621 |
| AM-MB-3 | 78001 | 34831 | 73934 | 0 | 4067 | 488 |
| MB-1 | 114086 | 42960 | 109858 | 0 | 4228 | 606 |
| MB-2 | 89524 | 34167 | 86687 | 0 | 2837 | 522 |
| MB-3 | 192880 | 68566 | 186182 | 0 | 6698 | 617 |
| AC-MB-3 | 93442 | 50306 | 88087 | 0 | 5355 | 1228 |
| AC-MB-1 | 88685 | 47034 | 84690 | 0 | 3995 | 1173 |
| AC-MB-2 | 95775 | 56052 | 91254 | 0 | 4521 | 1261 |
| AC-CB-1 | 91372 | 37860 | 88360 | 0 | 3012 | 528 |
| AC-CB-2 | 107800 | 43314 | 105188 | 0 | 2612 | 474 |
| AC-CB-3 | 104914 | 40950 | 101640 | 0 | 3274 | 370 |
| CB-1 | 125339 | 50842 | 120401 | 0 | 4938 | 489 |
| CB-2 | 106181 | 45072 | 99646 | 0 | 6535 | 532 |
| CB-3 | 111444 | 44755 | 107377 | 0 | 4067 | 542 |
| Avg | 100587 | 49201 | 94288 | 0 | 6298 | 858 |

**Table S3. Alpha diversity of the studied bee bread and midgut samples. MB (MB-1, MB-2, MB-3)**: Bee bread samples made by *Apis mellifera*. **CB (CB-1, CB-2, CB-3):** Bee bread samples made by *Apis cerana*. **AM-MB (AM-MB-1, AM-MB-2, AM-MB-3)**: Midgut samples of *Apis mellifera* bees fed *Apis mellifera* bee bread. **AC-CB (AC-CB-1, AC-CB-2, AC-CB-3)**: Midgut samples of *Apis cerana* bees fed *Apis cerana* bee bread. **AM-CB (AM-CB-1, AM-CB-2, AM-CB-3)**: Midgut samples of *Apis mellifera* bees fed *Apis cerana* bee bread. **AC-MB (AC-MB-1, AC-MB-2, AC-MB-3)**: Midgut samples of *Apis cerana* bees fed *Apis mellifera* bee bread.

| **Sample** | **OTUs** | **Shannon** | **Chao1** | **Ace** | **Goods coverage** |
| --- | --- | --- | --- | --- | --- |
| MB-1 | 171 | 5.96 | 230.09 | 240.40 | 0.96 |
| MB-2 | 131 | 5.84 | 189.24 | 181.23 | 0.96 |
| MB-3 | 146 | 5.28 | 207.60 | 221.36 | 0.96 |
| AM-MB-1 | 97 | 5.10 | 126.18 | 136.48 | 0.98 |
| AM-MB-2 | 123 | 5.38 | 187.69 | 180.44 | 0.96 |
| AM-MB-3 | 117 | 5.30 | 150.91 | 169.30 | 0.98 |
| AM-CB-1 | 497 | 3.00 | 618.40 | 644.77 | 1.00 |
| AM-CB-2 | 486 | 3.40 | 710.38 | 694.13 | 1.00 |
| AM-CB-3 | 385 | 3.06 | 562.52 | 572.13 | 1.00 |
| CB-1 | 411 | 3.28 | 627.26 | 684.40 | 1.00 |
| CB-2 | 403 | 2.63 | 604.40 | 632.07 | 1.00 |
| CB-3 | 289 | 2.59 | 411.10 | 420.52 | 1.00 |
| AC-CB-1 | 1171 | 5.94 | 1660.54 | 1678.24 | 0.99 |
| AC-CB-2 | 990 | 6.98 | 1644.87 | 1408.17 | 0.99 |
| AC-CB-3 | 1092 | 6.83 | 1763.61 | 1611.94 | 0.99 |
| AC-MB-1 | 1049 | 3.75 | 1535.39 | 1471.28 | 1.00 |
| AC-MB-2 | 1125 | 5.36 | 1548.83 | 1519.50 | 1.00 |
| AC-MB-3 | 1095 | 3.71 | 1493.86 | 1470.91 | 1.00 |

**Table S5. Comparative results of the relative abundances of the 5 most abundant bacterial phyla for the studied bee bread and midgut samples. MB**: Bee bread samples made by *Apis mellifera*. **CB**: Bee bread samples made by *Apis cerana*. **AM-MB**: Midgut samples of *Apis mellifera* bees fed *Apis mellifera* bee bread. **AC-CB**: Midgut samples of *Apis cerana* bees fed *Apis cerana* bee bread. **AM-CB**: Midgut samples of *Apis mellifera* bees fed *Apis cerana* bee bread. **AC-MB**: Midgut samples of *Apis cerana* bees fed *Apis mellifera* bee bread. Values are means ± SME (n=3). Statistical analysis was performed by Tukey’s HSD test, and different letters in the same column (a, b, c) indicate a significant difference at P<0.05.

| **Groups** | **Proteobacteria** | **Firmicutes** | **Bacteroidetes** | **Actinobacteria** | **Planctomycetes** |
| --- | --- | --- | --- | --- | --- |
| MB | 59.98±4.69a | 13.92±2.54a | 7.79±0.72ab | 11.62±1.40a | 1.66±1.47a |
| CB | 57.88±4.97a | 13.13±0.38a | 14.95±1.93ab | 10.1±1.87a | 2.12±1.58a |
| AM-MB | 86.99±2.45b | 10.44±2.59a | 0.31±0.04a | 1.97±0.36b | 0.00±0.00a |
| AC-CB | 56.43±6.30a | 17.22±9.94a | 20.25±6.49b | 6.02±2.95ab | 0.02±0.02a |
| AM-CB | 28.44±1.84b | 49.38±1.80b | 12.4±0.61ab | 6.58±0.90ab | 0.53±0.40a |
| AC-MB | 21.52±2.49b | 64.21±8.19b | 12.34±6.53ab | 0.91±0.31b | 0.17±0.08a |
| F | 33.272 | 17.182 | 3.073 | 7.171 | 1.044 |
| P | 0.000 | 0.000 | 0.052 | 0.003 | 0.436 |

**Table S7. PERMANOVA statistical tests performed with unweighted UniFrac distances and Bray-Curtis dissimilarity for diet and midgut microbial communities of *Apis mellifera* and *Apis cerana*. MB**: Bee bread samples made by *Apis mellifera*. **CB**: Bee bread samples made by *Apis cerana*. **AM-MB**: Midgut samples of *Apis mellifera* bees fed *Apis mellifera* bee bread. **AC-CB**: Midgut samples of *Apis cerana* bees fed *Apis cerana* bee bread. **AM-CB**: Midgut samples of *Apis mellifera* bees fed *Apis cerana* bee bread. **AC-MB**: Midgut samples of *Apis cerana* bees fed *Apis mellifera* bee bread. *P<0.05, and **P<0.01.

| **Metric** | **Variable** | **F** | **R²** | ***P*** | ***P*. signif** |
| --- | --- | --- | --- | --- | --- |
| Unweighted UniFrac | CB-vs-MB | 1.096 | 0.2151 | 0.3 | ns |
|  | CB-vs-AC-CB | 2.6998 | 0.403 | 0.1 | ns |
|  | MB-vs-AM-MB | 2.6361 | 0.3972 | 0.1 | ns |
|  | CB-vs-AM-CB | 3.7538 | 0.4841 | 0.1 | ns |
|  | MB-vs-AC-MB | 3.5091 | 0.4673 | 0.1 | ns |
|  | AC-CB-vs-AC-MB | 4.4409 | 0.5261 | 0.1 | ns |
|  | AM-CB-vs-AM-MB | 4.2857 | 0.5172 | 0.1 | ns |
|  | AM-MB-vs-AC-CB | 2.0145 | 0.3349 | 0.1 | ns |
|  | AC-MB-vs-AM-CB | 1.0841 | 0.2132 | 0.1 | ns |
|  | AC-CB-vs-AC-MB-vs-AM-CB-vs-AM-MB-vs-CB-vs-MB | 3.1388 | 0.5667 | 0.001 | ** |
|  | AC-CB-vs-AM-MB-vs-CB-vs-MB | 2.2826 | 0.4612 | 0.001 | ** |
|  | AC-CB-vs-AM-MB-vs-AC-MB-vs-AM-CB | 3.4748 | 0.5658 | 0.001 | ** |
|  | CB-vs-MB-vs-AC-MB-vs-AM-CB | 2.782 | 0.5106 | 0.001 | ** |
| Bray-Curtis | CB-vs-MB | 0.946 | 0.1913 | 0.6 | ns |
|  | CB-vs-AC-CB | 3.6545 | 0.4774 | 0.1 | ns |
|  | MB-vs-AM-MB | 5.6959 | 0.5875 | 0.1 | ns |
|  | CB-vs-AM-CB | 5.1515 | 0.5629 | 0.1 | ns |
|  | MB-vs-AC-MB | 10.0328 | 0.715 | 0.1 | ns |
|  | AC-CB-vs-AC-MB | 8.1976 | 0.6721 | 0.1 | ns |
|  | AM-CB-vs-AM-MB | 10.0753 | 0.7158 | 0.1 | ns |
|  | AM-MB-vs-AC-CB | 8.8102 | 0.6877 | 0.1 | ns |
|  | AC-MB-vs-AM-CB | 5.0842 | 0.5597 | 0.1 | ns |
|  | AC-CB-vs-AC-MB-vs-AM-CB-vs-AM-MB-vs-CB-vs-MB | 6.4787 | 0.7297 | 0.001 | ** |
|  | AC-CB-vs-AM-MB-vs-CB-vs-MB | 4.3594 | 0.6205 | 0.002 | ** |
|  | AC-CB-vs-AM-MB-vs-AC-MB-vs-AM-CB | 8.8834 | 0.7691 | 0.001 | ** |
|  | CB-vs-MB-vs-AC-MB-vs-AM-CB | 5.7659 | 0.6838 | 0.001 | ** |

**Table S8. Pearson's correlation analysis results between characteristic indexes of dietary habits and the most abundant bacterial genera of *Apis mellifera* and *Apis cerana*.** *P<0.05, and **P<0.01. *****

| **Characteristic indexes of dietary type** | **Species** | **Bacterial group (Genus level)** | **R^2^** | ***P*** | ***P.siginf*** |
| --- | --- | --- | --- | --- | --- |
| CP | *Apis cerana* | *Apibacter* | -0.794 | 0.059 | ns |
| CP | *Apis mellifera* | *Apibacter* | 0.686 | 0.132 | ns |
| CP | *Apis cerana* | *Bombella* | 0.942 | 0.005 | ** |
| CP | *Apis mellifera* | *Bombella* | -0.922 | 0.009 | ** |
| CP | *Apis cerana* | *Frischella* | -0.67 | 0.146 | ns |
| CP | *Apis mellifera* | *Frischella* | 0.91 | 0.012 | * |
| CP | *Apis cerana* | *Gilliamella* | -0.948 | 0.004 | ** |
| CP | *Apis mellifera* | *Gilliamella* | 0.629 | 0.18 | ns |
| CP | *Apis cerana* | *Lactobacillus* | 0.647 | 0.165 | ns |
| CP | *Apis mellifera* | *Lactobacillus* | -0.639 | 0.172 | ns |
| CP | *Apis cerana* | *Snodgrassella* | -0.313 | 0.546 | ns |
| CP | *Apis mellifera* | *Snodgrassella* | 0.882 | 0.02 | * |
| Moisture | *Apis cerana* | *Apibacter* | 0.885 | 0.019 | * |
| Moisture | *Apis mellifera* | *Apibacter* | -0.642 | 0.169 | ns |
| Moisture | *Apis cerana* | *Bombella* | -0.938 | 0.006 | ** |
| Moisture | *Apis mellifera* | *Bombella* | 0.98 | 0.001 | ** |
| Moisture | *Apis cerana* | *Frischella* | 0.488 | 0.326 | ns |
| Moisture | *Apis mellifera* | *Frischella* | -0.925 | 0.008 | ** |
| Moisture | *Apis cerana* | *Gilliamella* | 0.944 | 0.005 | ** |
| Moisture | *Apis mellifera* | *Gilliamella* | -0.736 | 0.095 | ns |
| Moisture | *Apis cerana* | *Lactobacillus* | -0.817 | 0.047 | * |
| Moisture | *Apis mellifera* | *Lactobacillus* | 0.809 | 0.051 | ns |
| Moisture | *Apis cerana* | *Snodgrassella* | 0.273 | 0.601 | ns |
| Moisture | *Apis mellifera* | *Snodgrassella* | -0.944 | 0.005 | ** |
| pH | *Apis cerana* | *Apibacter* | -0.909 | 0.012 | * |
| pH | *Apis mellifera* | *Apibacter* | 0.384 | 0.453 | ns |
| pH | *Apis cerana* | *Bombella* | 0.913 | 0.011 | * |
| pH | *Apis mellifera* | *Bombella* | -0.933 | 0.007 | ** |
| pH | *Apis cerana* | *Frischella* | -0.473 | 0.343 | ns |
| pH | *Apis mellifera* | *Frischella* | 0.733 | 0.097 | ns |
| pH | *Apis cerana* | *Gilliamella* | -0.89 | 0.017 | * |
| pH | *Apis mellifera* | *Gilliamella* | 0.764 | 0.077 | ns |
| pH | *Apis cerana* | *Lactobacillus* | 0.82 | 0.046 | * |
| pH | *Apis mellifera* | *Lactobacillus* | -0.617 | 0.192 | ns |
| pH | *Apis cerana* | *Snodgrassella* | -0.525 | 0.284 | ns |
| pH | *Apis mellifera* | *Snodgrassella* | 0.955 | 0.003 | ** |

***** The data we used in this table was the same as in Figure S3-S5.
